# Employing Young’s modulus and Debye temperature to calculate the elastic, thermodynamic and thermophysical properties of titanium oxynitrides

**DOI:** 10.1098/rsos.231797

**Published:** 2024-10-10

**Authors:** Anton Antonovych Kozma

**Affiliations:** ^1^ Department of Physical and Colloid Chemistry, Educational and Scientific Institute of Chemistry and Ecology, Uzhhorod National University, Uzhhorod 88000, Ukraine

**Keywords:** sound (elastic, strain, thermal, or phonon) wave velocities, elastic moduli, Gruneisen’s constant, isobaric and isochoric heat capacities, minimum thermal conductivity

## Abstract

The values of the shear *v*_s_ and longitudinal *v*_l_ wave velocities were calculated for 14 selected titanium oxynitrides TiN_*x*_O_*y*_ using the known values of Young’s modulus and Debye temperature. The errors Δ of the calculations did not exceed ±0.01%. It turned out that some TiN_*x*_O_*y*_ samples are able to compete with artificial diamonds in terms of *v*_l_ values and can potentially be used in acoustic resonators for intelligent chemical and biochemical sensors. A number of elastic, thermodynamic and thermophysical quantities were calculated, and graphical dependencies between them were plotted. The established correlations were used to develop two algorithms for predicting the properties of TiN_*x*_O_*y*_ alloys based on a single experimental parameter, namely the X-ray coefficient of thermal expansion or pycnometric density. The highest accuracy was shown by the method based on the experimental density, which allowed to estimate, with acceptable errors, the values of the shear *v*_s_ and mean *v*_m_ wave velocities (Δ = ±(1–5)%), the minimum thermal conductivity *λ*_min_ within the framework of the Cahill‒Pohl model (Δ = ±(0–3)%), the isobaric *C*_p_ and isochoric *C*_V_ heat capacities (Δ < 1%); while the known experimental methods and alternative models for determining these quantities are characterized by wider error intervals: Δ(*v*_s_) = ±(1–10)%, Δ(*λ*) = ±(1–10)% and Δ(*C*_p_) = ±(1–3)%.

## 1. Introduction

Titanium oxynitrides TiN_*x*_O_*y*_ are commercial materials for solar energy conversion processes [1–5], which can also be used in seawater desalinators [1], in electro- and photochemical hydrogen generation [5–9], in energy storage devices [9,10] (namely in the electrodes of lithium- [11,12], sodium- [12] and magnesium-ion [12] batteries and supercapacitors [13–15]), as anticorrosive coatings [16–18], as biocompatible coatings for vascular stents [19–22] and as components of implants [22,23]. Currently, research works [24–34] focus on the development of new methods of synthesis and on the explorations of various (microstructural, morphological, optical, catalytic, electrophysical, etc.) properties of TiN_*x*_O_*y*_ samples.

**Figure 1.**
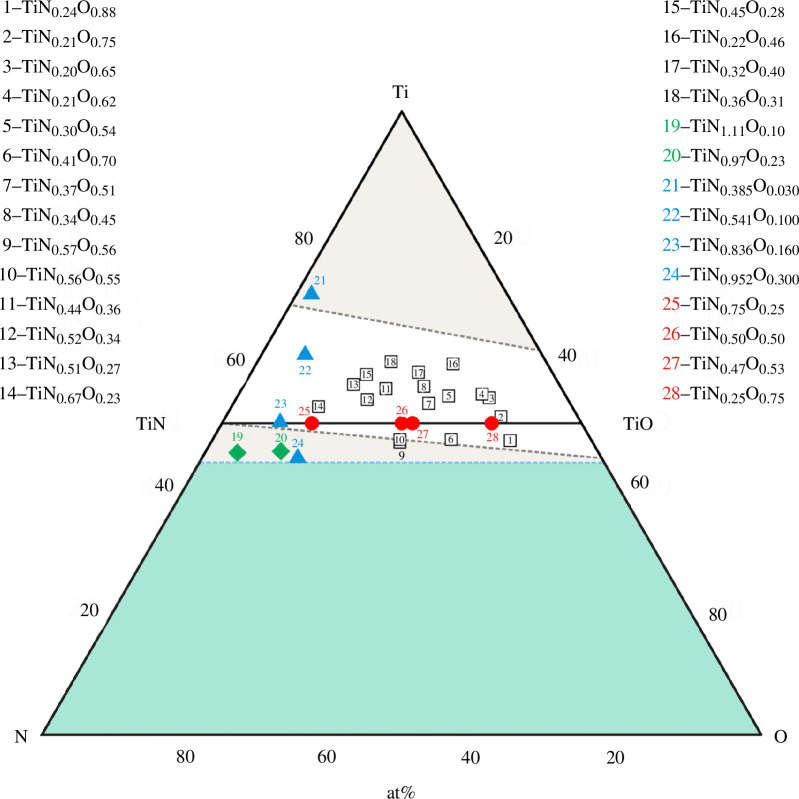
TiN_*x*_O_*y*_ alloys with known values of Young’s modulus or wave velocities in the Ti‒N‒O system: (i) samples numbered 1–18 (white squares) were taken from [35,36], 19 and 20 (green diamonds) from [38], 21–24 (blue triangles) from [39], 25–28 (red circles) from [40]; (ii) a homogeneous region is depicted with white colour, two heterogeneous regions are presented in grey, whereas the area not described in the literature is given in sea colour (the data are valid for sintering or annealing temperatures of 1473‒1773 K according to [45]).

Recent publications [9–23] show that in many cases, the prospect of practical use of titanium oxynitrides is related to their elastic characteristics. The use of these materials in various coatings, implants and energy storage devices could be more efficient if scientists had extensive data on the elastic properties of TiN_*x*_O_*y*_. Such information could also help to identify certain dependencies between the composition and elastic parameters. This would open up the possibility of targeted synthesis of titanium oxynitrides for more specific applications. However, the literature contains very little data on the elastic properties of TiN_*x*_O_*y*_. A literature survey covering the studies published in the past seven decades (from the 1950s to the present) has revealed only a few original works [35–40] reporting the Young’s modulus, one of the basic elastic parameters, for a small number of titanium oxynitrides (table 1) from small concentration regions of the ternary Ti‒N‒O system (figure 1).

**Table 1.** Known values of Young’s modulus *Е* and Debye temperature *θ*_D_ for titanium oxynitrides.

material	*E* (GPa)	*θ*_D_ (K)	material	*E* (GPa)	*θ*_D_ (K)
Alyamovsky *et al*. (1972) [35]					
TiN_0.21_O_0.75_	360	650	TiN_0.30_O_0.54_	350	620
Zainulin *et al*. (1977) [36]					
TiN_0.36_O_0.31_	340	575	TiN_0.20_O_0.65_	340	600
TiN_0.22_O_0.46_	350	590	TiN_0.37_O_0.51_	360	630
TiN_0.32_O_0.40_	350	595	TiN_0.52_O_0.34_	380	660
TiN_0.45_O_0.28_	370	625	TiN_0.67_O_0.23_	400	700
TiN_0.21_O_0.62_	340	600	TiN_0.24_O_0.88_	370	670
TiN_0.34_O_0.45_	340	600	TiN_0.41_O_0.70_	380	690
TiN_0.51_O_0.27_	370	630	TiN_0.56_O_0.55_	390	710
TiN_0.44_O_0.36_	375	635	TiN_0.57_O_0.56_	395	710
Zhang *et al*. (2009) [37]					
TiN_x_O_*y*_	185‒268	—	—	—	—
Do *et al*. (2013) [38]					
TiN_0.97_O_0.23_	400	—	TiN_1.11_O_0.10_	430	—
Goupy *et al*. (2013) [39]^a^					
TiN_0.385_O_0.03_	178	470	TiN_0.952_O_0.30_	193	440
TiN_0.541_O_0.10_	219	500	TiN_0.836_O_0.16_	—	—
Xiao *et al*. (2014) [40]^b^					
TiN_0.75_O_0.25_	376	—	TiN_0.47_O_0.53_ (with vacancy)	293	—
TiN_0.50_O_0.50_	270	—	TiN_0.25_O_0.75_	256	—

^a^ Calculated in this work by using simple formulas from [41–43], the experimental values of wave velocities and densities were taken from [39] and the values of fundamental constants were taken from [44].

^b^ The values were calculated in this work, based on the formulas and graphical data from [40].

Table 1 and figure 1 show that the Young’s modulus *Е* is known for 27 TiN_*x*_O_*y*_ samples (the data from [37] are not taken into account here, because the authors did not indicate the exact composition of the materials studied; also, the TiN_0.836_O_0.16_ alloy, for which only the longitudinal wave velocity was studied [39], is not taken into account). However, in the Ti‒N‒O system (figure 1), many hundreds of alloys can be formed, and among them there may be many samples with promising elastic or other properties.

In addition to the basic modulus *E*, it is important to consider other elastic parameters of titanium oxynitrides: the bulk modulus *B*, the shear modulus *G*, the Poisson ratio *σ* and the wave velocities—longitudinal *v*_l_, shear *v*_s_ or transverse *v*_t_, mean or root mean square *v*_m_. Note that in the literature, such waves propagating with certain velocities are called strain or elastic waves [43,46,47], acoustic or sound waves [43], phonons or thermal waves [47] (according to Debye [47], heat is conducted in a crystal lattice at the speed of phonons or sound). The available information on these properties of TiN_*x*_O_*y*_ is presented in table 2.

**Table 2.** Known and unknown elastic properties of titanium oxynitrides.

sample composition or concentration interval	number of samples examined	*B*	*G*	*σ*	*v*_l_	*v*_s_	*v*_m_
TiN_0.21_O_0.75_, TiN_0.30_O_0.54_ [35]	2	‒	‒	‒	‒	‒	‒
TiN_0.20‒0.67_O_0.23‒0.88_ [36]	16	‒	‒	‒	‒	‒	‒
TiN_0.97_O_0.23_, TiN_1.11_O_0.10_ [38]	2	‒	‒	‒	‒	‒	‒
TiN_0.385_O_0.03_, TiN_0.541_O_0.10_, TiN_0.952_O_0.30_ [39]	3	*	*	*	+	+	*
TiN_0.836_O_0.16_ [39]	1	‒	‒	‒	+	‒	‒
TiN_0.25‒0.75_O_0.25‒0.75_ [40]	4	+	+	+	‒	‒	‒

+, the property is known.

*, the property is not given in the original work [39], but can be calculated using simple formulas for the relationship between wave velocities, elastic constants *C*_*ij*_ and elastic moduli [39,40,43,46].

–, the property is unknown and cannot be calculated by simple formulas from [39,40,43,46].

As can be seen from table 2, for the found samples with mostly known values of *E*, the parameters *B*, *G* and *σ* have been studied for only four alloys [40]. For three more titanium oxynitrides from [39], these elastic properties can be calculated using classical simple formulas reported in [40,43,46]. An experimental study of the longitudinal wave velocities was carried out only for four TiN_*x*_O_*y*_ alloys with a small content of oxygen [39]; so one can state that even for the 28 titanium oxynitrides presented in the literature, the vast majority of elastic properties remain unknown. This scientific problem could be solved by solving the system of equation (1.1) for most TiN_*x*_O_*y*_ alloys:


(1.1) $$\left\{ \begin{array}{l} v_{l}={\left[\left(v_{s}^{2}E-4\rho v_{s}^{4}\right)/\left(E-3\rho v_{s}^{2}\right)\right]}^{\frac{1}{2}}\\ v_{l}={\left[4\pi V/\left(9N_{A}/{\left[\theta_{D}k_{B}/h_{P}\right]}^{3}-8\pi Vv_{s}^{-3}\right)\right]}^{\frac{1}{3}}, \end{array}\right.$$

where *ρ* is the density, *V* is the molar volume, *N*_A_ is the Avogadro constant, *k*_B_ is the Boltzmann constant and *h*_P_ is the Planck constant.

Note that the above system (1.1) is formed from the classical expressions (1.2) and (1.3) [41–43]:


(1.2) $$E=\rho v_{s}^{2}\left(3v_{l}^{2}-4v_{s}^{2}\right)/\left(v_{l}^{2}-v_{s}^{2}\right),$$

(1.3) $$\theta_{\text{D}}=h_{P}/k_{B}{\left[9N_{A}/\left(4\pi V\left[2v_{s}^{-3}+v_{l}^{-3}\right]\right)\right]}^{\frac{1}{3}}.$$

The literature [35,36] contains the necessary values of the Debye temperature (table 1) and density for most of the samples listed in table 2 and belonging to the same concentration interval TiN_0.20‒0.67_O_0.23‒0.88_. Hence, it is possible to solve the system of equation (1.1) and determine the unknown values of *v*_s_ and *v*_l_. In turn, this may allow us to apply the simplified expressions from [39,40,43] to estimate many other elastic properties of TiN_*x*_O_*y*_. In addition, the availability of the wave velocities or elastic constants sometimes allows us to determine the thermal properties [46,48], which have been studied for titanium oxynitrides even less systematically than the elastic parameters.

It should be noted that solving the system of equation (1.1) is a complex mathematical problem that requires the use of modern computing capabilities and has not been solved for TiN_*x*_O_*y*_ alloys before.

Thus, the purpose of this work was to calculate many unknown elastic, thermodynamic and thermophysical properties for a number of titanium oxynitrides from the concentration range TiN_0.20‒0.67_O_0.23‒0.88_ by using the values of their Young’s modulus and Debye temperature given in the literature.

## 2. Methods

### 2.1. Initial data

For the calculations, 18 samples were selected from the concentration range TiN_0.20‒0.67_O_0.23‒0.88_.

The alloys were synthesized by sintering at 1773 K (holding at the maximum temperature lasted from 5 to 80 h) under high vacuum conditions of 0.01‒0.13 Pa followed by rapid cooling [45,49,50]. These conditions made it possible to stabilize the high-temperature phase composition of titanium oxynitrides, which was confirmed by X-ray studies [45,49,50]. The initial data for the calculations were the values of *E*, *θ*_D_ and *ρ* of these samples, which were taken from the works of Alyamovsky and co-authors [35,36].

Since the literature does not contain the density values for the two alloys TiN_0.21_O_0.75_ and TiN_0.30_O_0.54_, we used the values of 5180 and 5160 kg m^−3^, respectively, which were estimated in this work.

The values of the fundamental constants (*h*_P_, *k*_B_, *N*_A_ and *π*), as well as the atomic weights of the elements for calculating the molar masses of M(TiN_*x*_O_*y*_), were taken from [44].

### 2.2. Initial calculations

Modern publications [51–56] mostly use equation (2.1) [57] for titanium-containing materials consisting of atoms of two or more elements:


(2.1) $$\theta_{\text{D}}=\left(h_{P}/k_{B}\right){\left(3qN_{A}/4\pi V\right)}^{\frac{1}{3}}v_{m},$$

where *q* is the number of atoms in a molecule or formula unit.

In turn, the value of *v*_m_ from expression (2.1) can be calculated using equation (2.2) [57]:


(2.2) $$v_{m}={\left[\left(2/v_{s}^{3}+1/v_{l}^{3}\right)/3\right]}^{-\frac{1}{3}}.$$

Combining formulas (1.2), (2.1) and (2.2), we can obtain the system of equation (2.3):


(2.3) $$\left\{ \begin{array}{l} E\;=\;\rho v_{s}^{2}\left(3v_{l}^{2}-4v_{s}^{2}\right)/\left(v_{l}^{2}-v_{s}^{2}\right)\\ \theta_{\text{D}}\;=\;\left(h_{P}/k_{B}\right){\left(3qN_{A}/4\pi V\right)}^{\frac{1}{3}}{\left[\left(2/v_{s}^{3}+1/v_{l}^{3}\right)/3\right]}^{-\frac{1}{3}}. \end{array}\right.$$

In this paper, system (2.3) was transformed into expression (2.4) with one unknown value of *v*_s_:


(2.4) $${\left[\left(v_{s}^{2}E-4\rho v_{s}^{4}\right)/\left(E-3\rho v_{s}^{2}\right)\right]}^{\frac{1}{2}}\;=\;{\left[4\pi V\theta_{D}^{3}k_{B}^{3}/\left(9qN_{A}h_{P}^{3}-8\pi Vv_{s}^{-3}\theta_{D}^{3}k_{B}^{3}\right)\right]}^{\frac{1}{3}}.$$

Equation (2.4) was solved using WolframǀAlpha computational intelligence [58]. For each TiN_*x*_O_*y*_ sample, the computational resource [58] offered certain intervals of numerical solutions that contained from several hundred to several thousand possible solutions. All the ranges of numerical values could be grouped into three large groups: the first gave *v*_s_ < 0 and *v*_l_ < 0; the second gave *v*_s_ > 0 and *v*_l_ > 0 at *v*_s_ > *v*_l_; while the third gave *v*_s_ > 0 and *v*_l_ > 0 at *v*_s_ < *v*_l_. In this paper, the intervals of the first and second groups were considered erroneous and were not taken into account. This can be explained by the classical concepts of solid-state physics [43]: the wave velocities cannot be negative. In addition, in solids, *v*_s_ cannot be greater than *v*_l_ [43]. From the many solutions of the third group, we chose one value of the shear wave velocity (the best of the initial results). When checking the results, this *v*_s_ value led to the smallest differences between the calculated values of *E* and *θ*_D_ and the literature data taken from [35,36] (columns 2 and 3 in table 3).

**Table 3.** Deviations of the *E* and *θ*_D_ values calculated using equation (2.4) from the original *E* and *θ*_D_ data reported in [35,36].

sample	best of primary results		maximum error reduction of *E*		maximum error reduction of *θ*_D_		best attempt at joint errors reduction	
	Δ(*E*) (%)	Δ(*θ*_D_) (%)	Δ(*E*) (%)	Δ(*θ*_D_) (%)	Δ(*E*) (%)	Δ(*θ*_D_) (%)	Δ(*E*) (%)	Δ(*θ*_D_) (%)
TiN_0.24_O_0.88_	‒8.99	13.25	0.00	‒68.18	‒58.65	0.00	48.88	‒18.01
	*v*_s_ < v_l_		v_s_ > v_l_ (!)		v_s_ < v_l_		v_s_ > v_l_ (!)	
TiN_0.21_O_0.75_	‒7.14	14.36	0.00	‒75.89	‒55.56	0.00	44.58	‒19.11
	v_s_ < v_l_		v_s_ > v_l_ (!)		v_s_ < v_l_		v_s_ > v_l_ (!)	
TiN_0.20_O_0.65_	‒22.06	11.69	0.00	‒37.28	E < 0 (!)	‒0.08	59.96	‒13.97
	v_s_ < v_l_		v_s_ > v_l_ (!)		v_s_ < v_l_		v_s_ > v_l_ (!)	
TiN_0.21_O_0.62_	‒69.20	12.13	0.01	‒38.15	E < 0 (!)	0.00	60.17	‒14.08
	v_s_ < v_l_		v_s_ > v_l_ (!)		v_s_ < v_l_		v_s_ > v_l_ (!)	
TiN_0.30_O_0.54_	‒18.29	11.74	0.00	‒47.28	‒86.43	0.00	59.38	‒15.26
	v_s_ < v_l_		v_s_ > v_l_ (!)		v_s_ < v_l_		v_s_ > v_l_ (!)	
TiN_0.41_O_0.70_	0.00	14.36	—		‒40.58	‒0.01	‒8.30	9.72
	v_s_ < v_l_				v_s_ < v_l_		v_s_ < v_l_	
TiN_0.37_O_0.51_	‒18.89	11.71	0.00	‒45.14	‒34.04	0.00	60.04	‒14.98
	v_s_ < v_l_		v_s_ >v_l_ (!)		v_s_ < v_l_		v_s_ > v_l_ (!)	
TiN_0.34_O_0.45_	‒21.23	11.61	0.01	‒49.56	E < 0 (!)	0.01	60.36	‒29.30
	v_s_ < v_l_		v_s_ > v_l_ (!)		v_s_ < v_l_		v_s_ > v_l_ (!)	
TiN_0.57_O_0.56_	0.00	14.15	—		‒37.75	0.00	‒10.76	8.80
	v_s_ < v_l_				v_s_ < v_l_		v_s_ < v_l_	
TiN_0.56_O_0.55_	0.00	13.89	—		‒33.89	0.00	‒12.97	8.04
	v_s_ < v_l_				v_s_ < v_l_		v_s_ < v_l_	
TiN_0.44_O_0.36_	‒39.40	13.19	0.00	43.53	E < 0 (!)	0.00	57.01	‒14.88
	v_s_ < v_l_		v_s_ > v_l_ (!)		v_s_ < v_l_		v_s_ > v_l_ (!)	
TiN_0.52_O_0.34_	‒15.21	11.89	0.00	‒62.96	‒62.21	0.01	51.98	‒17.26
	v_s_ < v_l_		v_s_ > v_l_ (!)		v_s_ < v_l_		v_s_ > v_l_ (!)	
TiN_0.51_O_0.27_	‒11.06	13.84	0.01	‒58.68	E < 0 (!)	0.00	60.57	‒14.50
	v_s_ < v_l_		v_s_ > v_l_ (!)		v_s_ < v_l_		v_s_ > v_l_ (!)	
TiN_0.67_O_0.23_	0.00	14.24	—		‒39.28	0.01	‒10.10	9.14
	v_s_ < v_l_				v_s_ < v_l_		v_s_ < v_l_	
TiN_0.45_O_0.28_	‒20.60	11.62	0.00	‒40.63	E<0 (!)	‒0.04	59.77	‒13.63
	v_s_ < v_l_		v_s_ > v_l_ (!)		v_s_ < v_l_		v_s_ > v_l_ (!)	
TiN_0.22_O_0.46_	‒70.29	0.00	0.00	‒33.36	—		149.87	‒11.43
	v_s_ < v_l_		v_s_ > v_l_ (!)				v_s_ > v_l_ (!)	
TiN_0.32_O_0.40_	0.00	23.61	—		E < 0 (!)	0.01	‒11.56	17.31
	v_s_ < v_l_				v_s_ < v_l_		v_s_ < v_l_	
TiN_0.36_O_0.31_	0.00	25.09	—		E < 0 (!)	‒0.01	‒8.93	19.56
	v_s_ < v_l_				v_s_ < v_l_		v_s_ < v_l_	

(!), implausible inequalities *v*_s_ > *v*_l_ and *Е <* 0, which contradict the principles of solid-state physics.

It should be noted that even the best of the initial results were characterized by rather high deviations (errors) Δ ≤ 70% (columns 2 and 3 in table 3). Thus, for some samples, the values of the Young’s modulus were overestimated by 246 GPa, which corresponded to the errors in the range of 0.00‒70.29%. At the same time, the Δ values for the Debye temperature varied within a narrower range of 0.00‒25.09% and led to the maximum deviations of ≤144 K. Unfortunately, such results could not be considered satisfactory and the calculations needed to be refined. For this purpose, the optimal value of the shear wave velocity was searched for each sample. The selection step was 1 m s^−1^. It was expected that the true values of *v*_s_(TiN_*x*_O_*y*_) would be in the range from 3500 to 7100 m s^−1^—this is the range of shear wave velocities of the binary initial components TiN [59–68] and TiO [40,69,70] and known titanium oxynitrides [39] (most of the literature values of *v_s_* from [40,59–69] were estimated using the classical ratio (*G*/*ρ*)^½^ [43]). However, in order to avoid additional errors, we studied a wider range of 300‒12 000 m s^−1^. Here we must note that sound wave velocities with the values of ~300 m s^−1^ can be considered as the minimum possible for solids and more typical for gaseous substances [44]. At the same time, the material with the record value of shear wave velocity (~12 000 m s^−1^ [71]) is diamond [71,72]. Thus, for solids, the acoustic parameter *v*_s_ should most likely be in the range of 300‒12 000 m s^−1^. Nevertheless, we did not exclude the possibility of finding optimal values of *v*_s_(TiN_*x*_O_*y*_) even beyond this range. Such an analysis of a wider range of wave velocities would make sense if the calculated errors decreased as we approached the limit values. However, we did not observe such features.

It should be noted that the optimizations led to an unexpectedly negative effect. The choice of the *v*_s_(TiN_*x*_O_*y*_) values that minimized the calculation errors of the *E* values led to a rapid increase in Δ(*θ*_D_) and to an abnormal decrease in the longitudinal wave velocities. At the same time, for 12 samples, the inequality *v*_s_ > *v*_l_ (!) was observed, which contradicted the basic principles of solid-state physics [43] (columns 4 and 5 of table 3).

Optimization of *v*_s_(TiN_*x*_O_*y*_) in the opposite direction led to the opposite effect: a decrease in Δ(*θ*_D_) was accompanied by a rapid increase in Δ(*E*). When checking the results for eight samples, even implausible negative values of the Young’s modulus were obtained (columns 6 and 7 in table 3).

The best attempt to jointly reduce the errors for *E* and *θ*_D_ (we used intermediate values of *v*_s_ and *v*_l_ rather than those used in the previous calculations for columns 4, 5 and 6, 7) also did not improve the accuracy of the calculations (columns 8 and 9 in table 3). For 12 samples, an anomalous inequality *v*_s_ > *v*_l_ (!) was observed. At the same time, for 6 alloys, it was possible to slightly reduce Δ(*θ*_D_) from the range of 13.89‒25.09% to the values in the range of 8.04‒19.56%. However, this led to an increase in Δ(*E*) from 0.00% to 8.30‒12.97% (columns 8 and 9 in table 3).

Thus, equation (2.4), which logically follows from traditional approaches [43,57], did not provide a single solution with a more or less acceptable error (≤ 5%) for any of the 18 TiN_*x*_O_*y*_ alloys selected for calculation.

### 2.3. Alternative calculations

In order to find a way to improve the accuracy of calculations, some alternative approaches were clearly required. Sometimes, to calculate the Debye temperature of complex materials, scientists [41] used a slightly simpler formula (1.3). In this case, the relationship between *E* and *θ*_D_ with wave velocities would correspond to the basic system of equation (1.1). In turn, system (1.1) can be transformed into expression (2.5) with one unknown parameter *v*_s_:


(2.5) $${\left[\left(v_{s}^{2}E-4\rho v_{s}^{4}\right)/\left(E-3\rho v_{s}^{2}\right)\right]}^{\frac{1}{2}}\;=\;{\left[4\pi V\theta_{D}^{3}k_{B}^{3}/\left(9N_{A}h_{P}^{3}-8\pi Vv_{s}^{-3}\theta_{D}^{3}k_{B}^{3}\right)\right]}^{\frac{1}{3}}.$$

Equation (2.5) was also solved using WolframǀAlpha computational intelligence [58]. The initial results are shown in table 4.

**Table 4.** Deviations of the *E* and *θ*_D_ values calculated using equation (2.5) from the original *E* and *θ*_D_ data reported in [35,36].

sample	primary results			results of optimization of values *v*_s_		
	Δ(*E*) (%)	Δ(*θ*_D_) (%)	*v*_s_ : *v*_l_	Δ(*E*) (%)	Δ(*θ*_D_) (%)	*v*_s_ : *v*_l_
TiN_0.24_O_0.88_	0.00	9.47	*v*_s_ < *v*_l_	0.00	0.00	*v*_s_ < *v*_l_
TiN_0.21_O_0.75_	0.00	9.63		0.00	0.00	
TiN_0.20_O_0.65_	‒0.01	10.37		0.00	0.01	
TiN_0.21_O_0.62_	0.00	10.39		‒0.01	0.01	
TiN_0.30_O_0.54_	0.00	10.10		0.00	0.00	
TiN_0.41_O_0.70_	‒0.01	9.36		0.00	0.00	
TiN_0.37_O_0.51_	0.00	10.06		0.00	0.00	
TiN_0.34_O_0.45_	0.01	10.49		0.00	‒0.01	
TiN_0.57_O_0.56_	0.00	9.28		0.00	0.00	
TiN_0.56_O_0.55_	‒0.01	9.26		0.00	0.00	
TiN_0.44_O_0.36_	0.00	10.34		0.00	0.00	
TiN_0.52_O_0.34_	‒0.01	9.87		0.00	0.00	
TiN_0.51_O_0.27_	0.00	10.42		0.00	0.00	
TiN_0.67_O_0.23_	‒0.01	9.53		0.00	0.00	
TiN_0.45_O_0.28_	0.00	10.61		‒0.65	0.65	
TiN_0.22_O_0.46_	0.00	11.26		‒2.73	2.73	
TiN_0.32_O_0.40_	0.00	11.03		‒2.95	2.95	
TiN_0.36_O_0.31_	0.00	11.69		‒4.94	4.94	

As seen, the error values have significantly decreased compared with the results in table 3. The values of Δ(*E*) reached the lowest possible values of 0.00‒0.01% (column 2 in table 4). At the same time, the errors in the Debye temperatures, although still quite high at 9.26‒11.69% (column 3 in table 4), were already in a narrower range of values. For all the results, the required inequality *v*_s_ < *v*_l_ was fulfilled (column 4 in table 4).

The subsequent optimization of the calculations was carried out similarly to §2.2. The initial values of *v*_s_ obtained using [58] were refined until the best agreements between the calculated *E* and *θ*_D_ values and the literature data [35,36] were achieved. The step of the refining optimization was also 1 m s^−1^.

As a result of our calculations for 14 TiN_*x*_O_*y*_ samples, we managed to achieve an almost absolute agreement (with an accuracy of 99.99–100.00% or with errors ≤0.01%) between the known *E* and *θ*_D_ [35,36] and the relevant values calculated in this work (columns 5 and 6 in table 4). For four more samples of titanium oxynitrides, the maximum errors could not be reduced below the level of 0.65–4.94% (the last four positions in columns 5 and 6 in table 4). These four worst results can also be considered satisfactory, since the largest error for them did not exceed 5%. The determined values of *v*_s_(TiN_*x*_O_*y*_) made it possible to calculate the corresponding values of *v*_l_ for all 18 samples using the system of equation (1.1). None of the optimized results contradicted the necessary requirement of solid- state physics, *v*_s_ < *v*_l_ (column 7 in table 4). Note also that all the optimized values of *v*_s_(TiN_*x*_O_*y*_) were in the range of 4752‒6012 m s^−1^; and this range falls within the expected values of *v*_s_ = 3500‒7100 m s^−1^ (see §2.2). It is also important to note that in the tested range of 300‒12000 m s^−1^, a single *v*_s_ value was recorded for each of the 18 alloys, which gave an optimal agreement between the initial parameters *E* and *θ*_D_. That is, no sample had several variants of the *v*_s_ value from different parts of the 300‒12 000 m s^−1^ range that could be consistent with the literature values of the Young’s modulus and Debye temperature [35,36] (table 1). Moreover, for each alloy, even a minimal shift (±1 m s^−1^) in the shear wave velocity led to a slight but noticeable lowering of the result accuracy. The applicability of the calculated *v*_s_ and *v*_l_ values in order to achieve the optimal agreement between *E* and *θ*_D_ is illustrated in appendix A.

### 2.4. The following calculations

For the 14 samples for which the calculation errors did not exceed 0.01% (table 4), the following calculations were performed. The average wave velocity was determined by expression (2.2). The values of the shear modulus *G*, bulk modulus *B* and Poisson ratio *σ* were calculated using formulas (2.6) [40,43], (2.7) [39,40,43,70], and (2.8) [39,46], respectively:


(2.6) $$G\;=\;v_{s}^{2}\rho \;=\;\frac{1}{2}\left[\left(\left[C_{11}-C_{12}+3C_{44}\right]/5\right)+\left(5C_{44}\left[C_{11}-C_{12}\right]/\left[4C_{44}+3\left(C_{11}-C_{12}\right)\right]\right)\right],$$

(2.7) $$B\;=\;v_{l}^{2}\rho -4v_{s}^{2}\rho /3\;=\;\left(C_{11}+2C_{12}\right)/3,$$

(2.8) $$\sigma \;=\;\left(v_{l}^{2}-2v_{s}^{2}\right)/2\left(v_{l}^{2}-v_{s}^{2}\right)\;=\;C_{12}/\left(C_{11}+C_{12}\right),$$

where *C*_11_, *C*_12_ and *C*_44_ are the elastic constants *C_ij_*.

## 3. Results and discussion

### 3.1. Elastic properties of TiN_*x*_O_*y*_

The calculated values of the elastic properties of titanium oxynitrides are collected in table 5. The TiN_*x*_O_*y*_ samples are arranged in ascending order of the nitrogen-to-oxygen (*x*/*y*) ratio in their composition.

**Table 5.** Calculated elastic properties of TiN_*x*_O_*y*_ samples stabilized at 1773 K^a^.

no.	material	*x*/*y*	*v*_s_ (m s^−1^)	*v*_l_ (m s^−1^)	*v*_m_ (m s^−1^)	*G* (GPa)	*B* (GPa)	*σ*
	
1	TiN_0.24_O_0.88_	0.2727	5736	8805	6289	164	167	0.131
2	TiN_0.21_O_0.75_	0.2800	5380	8790	5940	150	200	0.200
3	TiN_0.20_O_0.65_	0.3077	4799	17 394	5474	117	1376	0.459
4	TiN_0.21_O_0.62_	0.3387	4781	18 433	5457	116	1500 <	0.464
5	TiN_0.30_O_0.54_	0.5556	4975	10 960	5609	128	450	0.370
6	TiN_0.41_O_0.70_	0.5857	5868	8696	6405	176	152	0.082
7	TiN_0.37_O_0.51_	0.7255	5090	10 715	5726	133	412	0.354
8	TiN_0.34_O_0.45_	0.7556	4752	30 795	5436	114	1500 <	0.488
9	TiN_0.57_O_0.56_	1.0179	6012	8729	6544	188	146	0.049
10	TiN_0.56_O_0.55_	1.0182	6012	8673	6538	188	141	0.038
11	TiN_0.44_O_0.36_	1.2222	5030	16 307	5730	130	1189	0.447
12	TiN_0.52_O_0.34_	1.5294	5351	9774	5966	148	296	0.286
13	TiN_0.51_O_0.27_	1.8889	4978	20 792	5685	126	1500<	0.470
14	TiN_0.67_O_0.23_	2.9130	5747	9051	6320	172	197	0.162

^a^ The conditions of heat treatment of the samples are given in §2.1.

The data obtained (table 5) show that the elastic parameters of titanium oxynitrides strongly depend on their chemical composition. For example, for different *x*/*y* ratios, the values of *v*_l_, *B* and *σ* vary widely from ~0.5% to more than several hundred per cent. In particular, the difference between alloys, for example, no. 10(TiN_0.56_O_0.55_) and no. 8(TiN_0.34_O_0.45_), reaches 3.5 times in terms of the longitudinal wave velocity. Some samples differ even more in terms of the values of the bulk modulus and Poisson ratio. At the same time, the difference between the values of *v*_s_, *v*_m_ and *G* is in much narrower ranges and mostly does not exceed several tens of per cent. It can be seen that the values of the three parameters *v*_l_, *B* and *σ* vary widely, while the values of the other three parameters *v*_s_, *v*_m_ and *G* vary within much narrower limits. In addition, samples with high values of *v*_l_, *B* and *σ* mostly have low values of *v*_s_, *v*_m_ and *G*. In order to identify clearer correlations between these parameters, several dozen graphs were constructed, taking into account different variants of the dependencies between the elastic properties of TiN_*x*_O_*y*_ (table 6).

**Table 6.** The highest coefficient of determination *R* ^2^ values obtained in the mathematical processing of the graphical dependencies for the elastic properties of the TiN_*x*_O_*y*_ alloys.

*f*(*x*)		*v*_s_	*v*_l_	*v*_m_	*G*	*B*	*σ*
*f*(*y*)	*v*_s_	—	< 0.700	**0.996**	**0.994**	< 0.600	< 0.980
	*v*_l_	< 0.700	—	< 0.900	< 0.900	**0.999**	**0.984**
	*v*_m_	**0.996**	< 0.900	—	**0.988**	< 0.600	< 0.980
	*G*	**0.994**	< 0.900	**0.988**	—	< 0.800	< 0.980
	*B*	< 0.600	**0.999**	< 0.600	< 0.800	—	**0.990**
	σ	< 0.980	< 0.980	< 0.980	< 0.980	**0.996**	—

The promising values of *R* ^2^ > 0.980 are highlighted in bold.

Polynomial, exponential and sigmoidal equations were used to find the mathematical expressions which are close to the optimal ones. The expression was chosen based on the criterion of the best description of a given graphical dependence. If different equations gave the same value of the coefficient of determination *R* ^2^ [73], the simplest expression was preferred.

As a result, only for eight dependencies have high values of *R* ^2^ ≥ 0.990 (table 6) have been achieved. It should be noted that for each dependency, two graphs were plotted: *y = f*(*x*) and *x = f*(*y*), changing the elastic properties on the coordinate axes. This explains why table 6 contains 30 values of *R* ^2^ rather than the expected fifteen. This approach allowed us to achieve higher *R* ^2^ values for some dependencies. For example, for the curve *B = f*(*σ*), even the best fitted equations had the coefficient of determination values of only *R* ^2^ ≤ 0.990. At the same time, when mathematically processing the inverse relationship *σ = f*(*B*), we managed to achieve a higher value of *R^2^* = 0.996.

**Figure 2.**
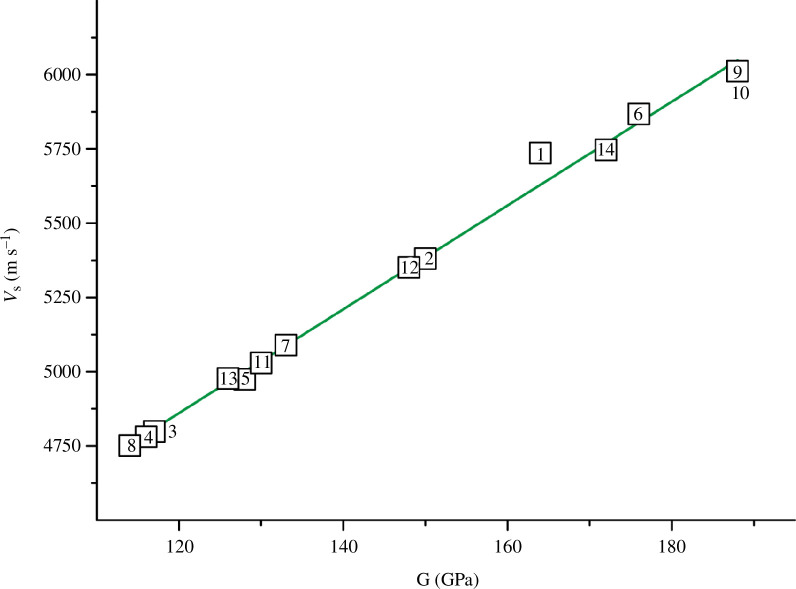
Dependence of the shear wave velocity on the shear modulus with *R* ^2^ = 0.994 for TiN_*x*_O_*y*_ (alloy numbers and composition are explained in figure 1 and table 5).

**Figure 3.**
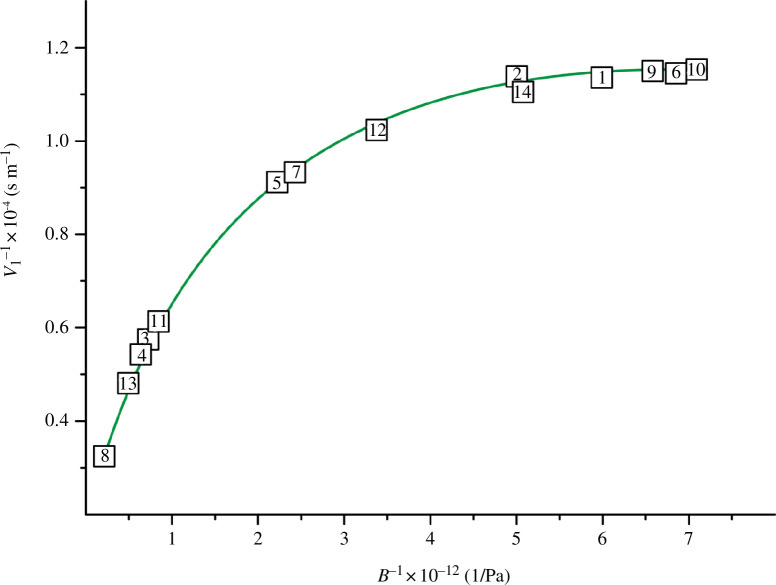
Dependence of 1/*v*_l_ on 1/*В* with *R* ^2^ = 0.999 for TiN_*x*_O_*y*_.

The noteworthy relationships between the *v*_s_, *v*_m_ and *G* values (conditionally referred to as the elastic properties of the first group) and between the *v*_l_, *B* and *σ* values (the elastic parameters of the second group) are mentioned in the description for table 5, but are more clearly revealed in table 6. The coefficient of determination values are high for the dependencies between the individual parameters of the first group (*v*_s_, *v*_m_, *G*), *R^2^* = 0.988‒0.996, and between the parameters of the second group (*v*_l_, *B*, *σ*), *R^2^* = 0.984‒0.999. However, for the cross-dependencies between the values of the first and second groups (*v*_s_, *v*_m_, *G* with *v*_l_, *B*, *σ*), the correlations are mostly low, *R^2^* = 0.500‒0.960. Let us look at some of the most illustrative examples in figures 2 and 3.

The dependencies in figures 2 and 3 are well described by equations (3.1) and (3.2):


(3.1) $$v_{s}=2762.973+17.478G,$$

(3.2) $$v_{l}=7497.491+7.975B-0.638\times 10^{-3}B^{2}.$$

According to expressions (3.1) and (3.2), the maximum differences from the initial values (see table 5) are within the narrow limits of ± 107 m s^−1^ for *v*_s_ and ± 277 m s^−1^ for *v*_l_. Due to this, the correlation formulas (3.1) and (3.2) lead to low calculated errors of ±(0.01‒3.06)%.

**Figure 4.**
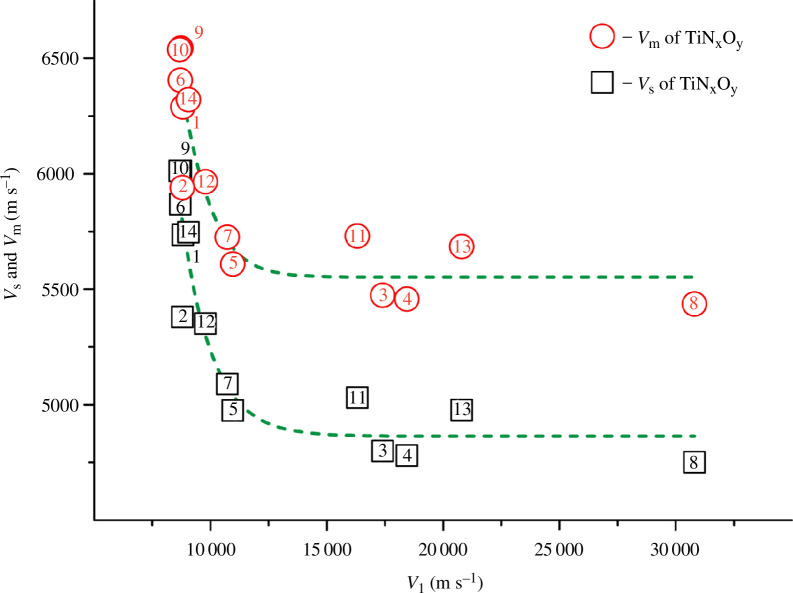
Dependence of the shear and mean wave velocities on the longitudinal wave velocity with *R* ^2^ = 0.670–0.860 for the TiN_*x*_O_*y*_ alloys.

In turn, for all the constructed dependencies between the properties from different groups (the first group: *v*_s_, *v*_m_, *G* and the second group: *v*_l_, *B*, *σ*), the correlations were weak. None of the fitted curves managed to achieve *R* ^2^ ≥ 0.990 (table 6). As an example, consider the graphical dependence between *v*_s_ and *v*_m_ (parameters from the first group) and *v*_l_ (the parameter from the second group) in figure 4.

A number of values (for alloys numbered 11, 3, 4, 13, 8) are scattered in wide range and do not fall on the same curve (figure 4). This did not allow us to obtain high coefficient of determination values for the dependencies *v*_s_ *= f*(*v*_l_) and *v*_m_ *= f*(*v*_l_). Even in the best cases, the *R* ^2^ values did not exceed 0.670–0.860.

The division of the elastic properties (table 5) into two conventional groups (*v*_s_, *v*_m_, *G* and *v*_l_, *B*, *σ*) can be explained using the classical formulas (2.2), (2.6)–(2.8) [39,40,43,46,57,70] (see §§2.2 and 2.4). According to expression (2.2), the mean wave velocities are more strongly influenced by the shear wave velocities rather than by the longitudinal ones. In particular, the values of *v*_m_ differ by only 9–14% from the corresponding values of *v*_s_ (table 5). In contrast, the differences between the *v*_m_ and *v*_l_ values range from ~30% to over 400% (table 5). In addition, the courses of the curves *v*_m_ *= f*(*v*_l_) and *v*_s_ *= f*(*v*_l_) are almost identical (figure 4). It should be noted that the shear wave velocity also directly affects the values of the shear modulus *G*, as can be seen from formula (2.6). This is additionally confirmed by the straight-line dependence on the *v*_s_ *= f*(*G*) graph (figure 2). At the same time, the shear modulus does not directly depend on *v*_l_ (expression (2.6)). Thus, the values of the first group (*v*_s_, *v*_m_, *G*) are strongly interrelated, and the parameters from the second group (*v*_l_, *B*, *σ*) have little effect on them.

A similar relationship exists between the properties of the second group (*v*_l_, *B*, *σ*). The longitudinal wave velocity *v*_l_ correlates best with the values of the bulk modulus *B* and the Poisson ratio *σ*. This is especially evident in the dependence *v*_l_ *= f*(*B*) (table 6, figure 3). A slightly lower degree of correlation (*R* ^2^ = 0.984) is observed for the curve *v*_l_ *= f*(*σ*). However, for the dependencies of *v*_l_ with elastic parameters of the first group (*v*_s_, *v*_m_, *G*), the indices of determination are lower (*R* ^2^ < 0.900). At the same time, the Poisson ratio correlates best with the volume modulus (*R* ^2^ = 0.996 in table 6).

Note that of the parameters of the first group, only the shear wave velocity has the greatest influence on the values of the second group *B* and *σ* (equations (2.7) and (2.8)). At the same time, *v*_s_ correlates best with the Poisson ratio. The corresponding coefficient of determination is close to ~0.960, but is not high enough for prognostic applications. The Poisson ratio correlates somewhat worse with the other values of the first group *v*_m_ and *G*, with *R* ^2^ = 0.930–0.950.

From formulas (1.2), (2.5), (2.6) and (2.7), it is also clear that the elastic properties of materials are significantly influenced by their density, which is one of the basic physical and chemical parameters. Hence, it is important to analyse the relationship between the elastic and basic physicochemical properties of titanium oxynitrides.

### 3.2. Relationship between elastic and physicochemical properties of TiN_*x*_O_*y*_

**Figure 5.**
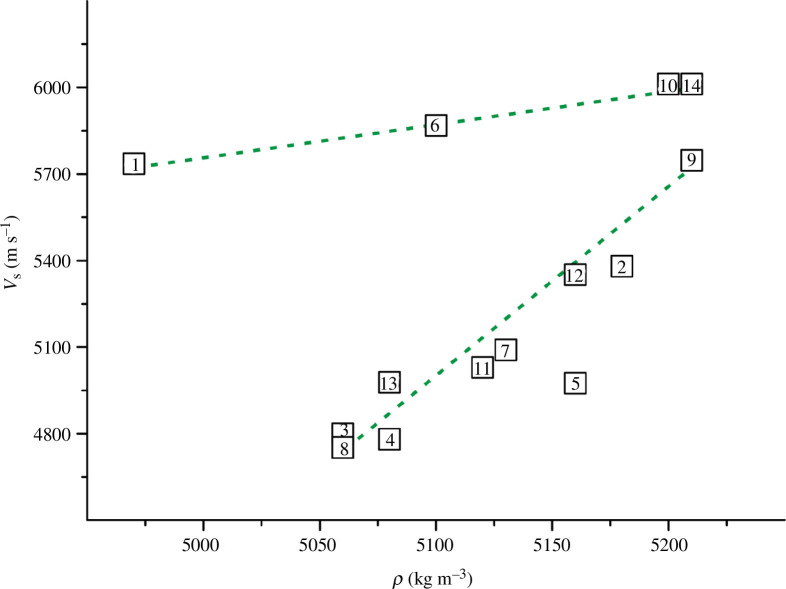
Dependence of shear wave velocity on density for TiN_*x*_O_*y*_ (values of *ρ* are taken from [36] or estimated in this work and presented in appendix A).

**Figure 6.**
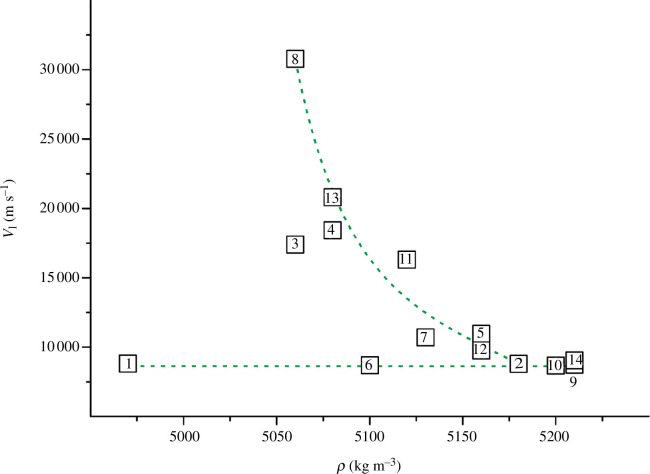
Dependence of longitudinal wave velocity on density for TiN_*x*_O_*y*_ (values of *ρ* are taken from [36] or estimated in this work and presented in appendix A).

Using the experimental [36,45] and estimated (see §2.1 and appendix A) values of *ρ* for 14 TiN_*x*_O_*y*_ alloys (table 5), we plotted the dependencies *v*_s_ *= f*(*ρ*) and *v*_l_ *= f*(*ρ*) in figure 5 and 6, respectively.

Figure 5 shows that for most alloys, *v*_s_ increases with increasing *ρ*. There are two close to linear dependencies between the two series of alloys (indicated by the green dashed line). For six alloys in figure 6 (numbered 1, 6, 2, 10, 9, 14), the longitudinal wave velocity is weakly dependent on *ρ*. For the remaining samples (numbered 8, 13, 4, 11, 7, 5, 12), the values of *v*_l_ decrease rapidly with increasing density. Thus, for the selected 14 TiN_*x*_O_*y*_ alloys, there is no single clear correlation between wave velocities and density.

Sometimes [74], in addition to the usual density *ρ* (mass density or specific gravity), the atomic density *p* is used to characterize the physicochemical properties of titanium-containing materials. Analysing the results of work [74] on the calculation of *p* values for titanium oxides Ti_*x*_O_y_, we came to equation (3.3):


(3.3) $$p\;=\;\left(\rho /M\right)qN_{A}.$$

The values required for expression (3.3) for selected titanium oxynitrides are given in appendix A.

**Figure 7.**
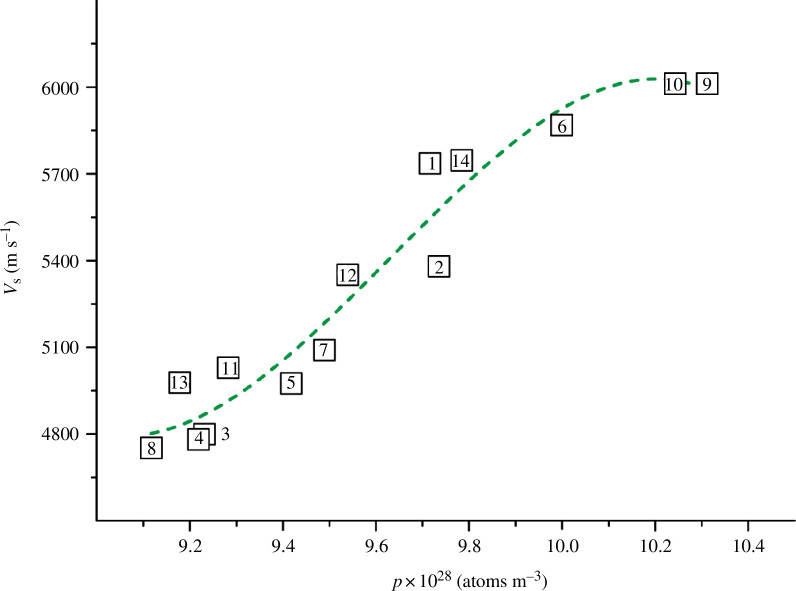
Dependence of the shear wave velocity on the atomic density for TiN_*x*_O_*y*_ (values of *p* were calculated using formula (3.3)).

**Figure 8.**
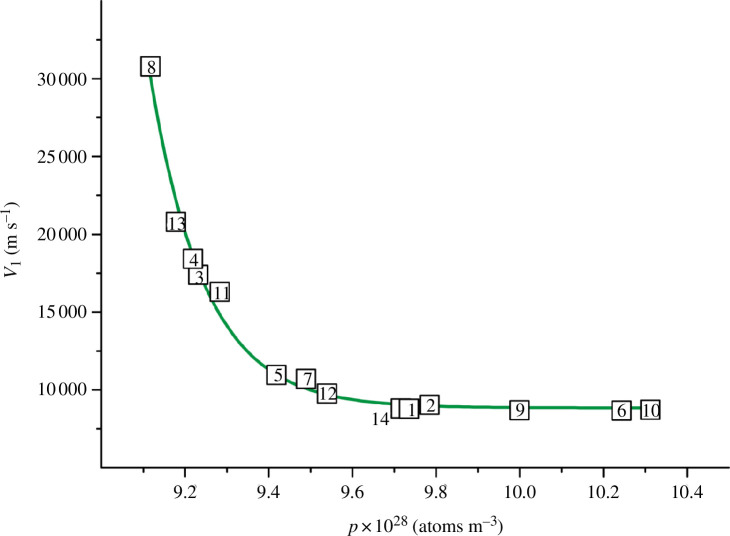
Dependence of the longitudinal wave velocity on the atomic density for TiN_*x*_O_*y*_ (values of *p* were calculated using formula (3.3)).

The constructed graphical dependencies *v*_s_ *= f*(*p*) and *v*_l_ *= f*(*p*) are shown in figures 7 and 8, respectively.

As can be seen from figures 7 and 8, the atomic density *p*, unlike the conventional density *ρ*, is clearly correlated with the shear and longitudinal wave velocities. The shear wave velocity increases with increasing values of *p* (figure 7). The opposite pattern is observed for the longitudinal wave velocity: the values of *v*_l_ decrease with increasing atomic density (figure 8).

For all 14 TiN_*x*_O_*y*_ alloys, the *p* values can be combined with wave velocities by the correlation dependencies that have rather high *R* ^2^ values. For the *v*_s_ *= f*(*p*) graph in figure 7, we managed to select an equation with a maximum value of the coefficient of determination of ~0.930. At the same time, for the dependence *v*_l_ *= f*(*p*) in figure 8, the expression close to the optimal one had a higher value, *R* ^2^ = 0.988. The corresponding equation is as follows:


(3.4) $$v_{l}=8839.840+\left\{51151160.160/\left(1+{exp}^{\left(p-8.096\right)/0.131}\right)\right\},$$

where exp is the exponent.

Note that to simplify the calculations, formula (3.4) does not take into account the power factor (28 for the number 10^28^) of the atomic density. Therefore, for all the alloys in table 5, we took the *p* values from the range 9.117‒10.312, rather than the actual values (9.117‒10.312) × 10^28^ atoms m^−3^.

The verification of expression (3.4) showed that the errors of the calculated values of *v*_l_ for 11 alloys fluctuated within acceptable limits of ±(0.0‒2.9)%. However, for three samples, the deviation was more significant, from 6% to ~9%, which gave a maximum error of ≤1530 m s^−1^ for *v*_l_ values. Apparently, formula (3.4) will allow predicting longitudinal wave velocities for other poorly studied TiN_*x*_O_*y*_ alloys with an accuracy of 91–100%. Then, for new samples, it will be sufficient to determine the experimental (pycnometric) density, and then use equations (3.3) and (3.4) to approximate the values of *v*_l_.

**Figure 9.**
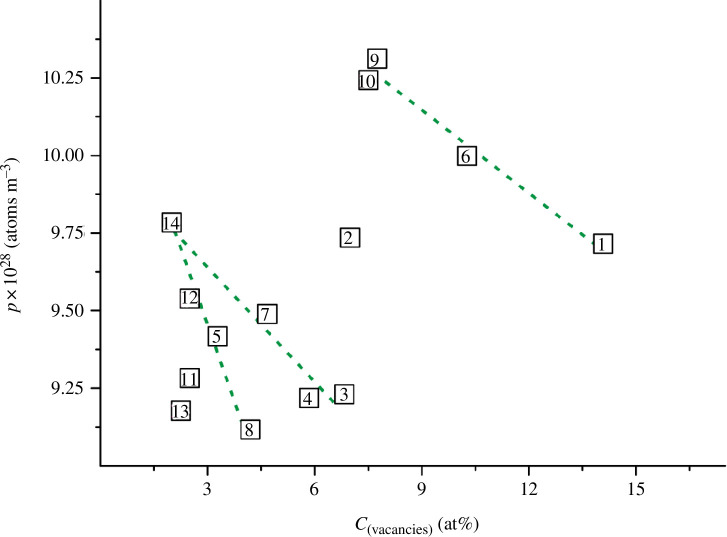
Dependence of the atomic density on the vacancy concentration for TiN_*x*_O_*y*_ (values of *p* were calculated using formula (3.3) ; values of*C*_(vacancies)_ were obtained as a result of simple mathematical processing of the data from [35,36,45]).

**Figure 10.**
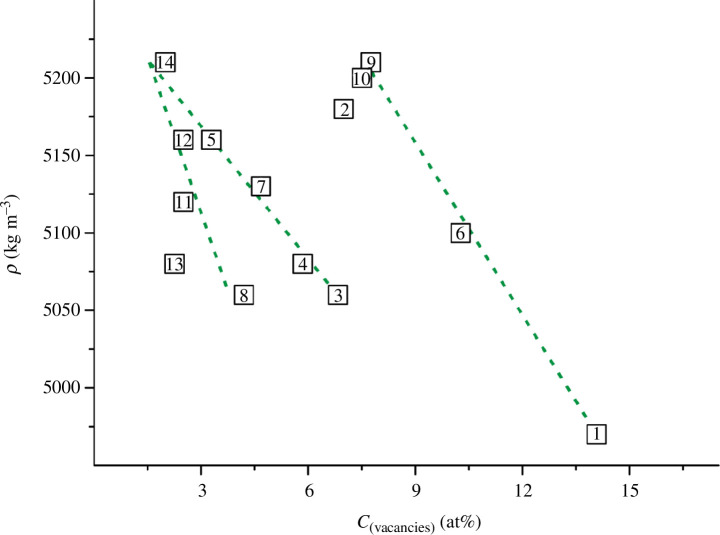
Dependence of density on vacancy concentration for TiN_*x*_O_*y*_ (values of *ρ* are taken from [36] or estimated in this work and presented in appendix A; values of *C*_(vacancies)_ were obtained as a result of simple mathematical processing of the data from [35,36,45]).

It can be assumed that in titanium oxynitride alloys, the atomic density decreases owing to an increase in the number of atomic vacancies. According to [36], TiN_*x*_O_*y*_ alloys are characterized by the presence of such structural defects. It should be noted that the starting components for the synthesis of titanium oxynitrides are mainly TiN and TiO [49,75], which also have atomic vacancies [76,77]. Thus, titanium mononitride has up to 2% [76] or up to 4% [77] of such defects, while titanium monooxide up to 15–17% [76,77]. In order to verify the above assumption, we calculated the concentration of atomic vacancies *C*_(vacancies)_ for all TiN_*x*_O_*y*_ alloys from table 5. The values of *C*_(vacancies)_ was determined by the difference between the theoretical (X-ray) and experimental (pycnometric) densities. The theoretical density was calculated using the molar mass (see appendix A) and the lattice constant *a*, the values of which were taken from [35,36]. It was assumed that the unit cell of each TiN_*x*_O_*y*_ alloy should contain four formulaic units (this follows from the presence of eight atoms in the unit cells of the original TiN [78] and TiO [79]). The values of the experimental densities required to construct the graphical dependencies were taken from [36,45] or estimated in this work (see appendix A). As a result, it turned out that each of the 14 samples from table 5 has from 2% to 14% of atomic vacancies. Such values can be considered commensurate or intermediate compared to individual TiN and TiO [76,77]. In order to identify certain regularities, we plotted the dependencies *p = f*(*C*_(vacancies)_) in figure 9 and *ρ = f*(*C*_(vacancies)_) in figure 10.

**Figure 11.**
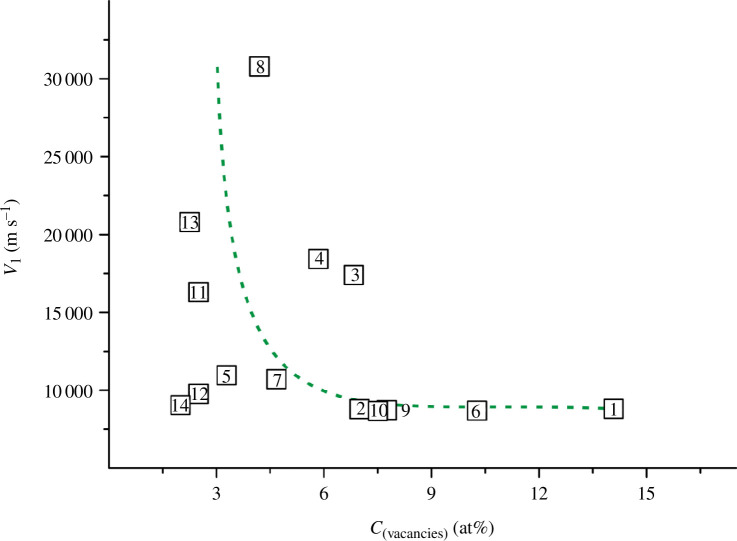
Dependence of the longitudinal wave velocity on the vacancy concentration for TiN_*x*_O_*y*_ (values of *C*_(vacancies)_ were obtained as a result of simple mathematical processing of the data from [35,36,45]).

**Figure 12.**
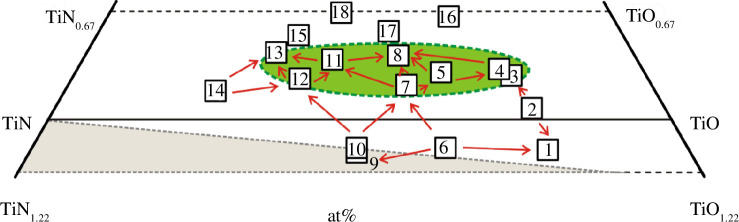
Concentration region of TiN_1.22_‒TiN_0.67_‒TiO_0.67_‒TiO_1.22_ with the wave velocities calculated in this work for TiN_*x*_O_*y*_ alloys: (i) arrows show the directions of growth of *v*_l_; (ii) the area of alloys with the highest *v*_l_ values is highlighted in green; (iii) the homogeneous region is highlighted in white and green and the heterogeneous region is grey at sintering temperatures of 1473‒1773 K (based on the review data from [45]).

Figures 9 and 10 show that for titanium oxynitrides there is no unambiguous correlation between different densities (*p* and *ρ*) and the vacancy concentration. We can only identify the scattered linear dependencies for individual groups of 4‒5 alloys. For example, in both graphs (figures 9 and 10), the most obvious is the grouping of samples by numbers 10, 9, 6, 1, for which a decrease in the values of *p* and *ρ* is observed with an increase in the values of *C*_(vacancies)_. For these four alloys, the concentration of vacancies increases linearly in the direction of increasing content of titanium monoxide. This dependence is actually expected, since the initial component TiO (not TiN) is more prone to the formation of structural vacancies [76,77]. By a similar analogy, two more groups of alloys can be distinguished: 14, 7, 4, 3 (figure 9) or 14, 5, 7, 4, 3 (figure 10) and 14, 12, 5, 8 (figure 9) or 14, 12, 11, 8 (figure 10). It should be noted that some samples can only be conditionally assigned to one group of alloys. In particular, sample no. 5 fits differently on different graphs. Nevertheless, for most of the alloys from the selected groups, the values of *p* and *ρ* monotonically decrease in the direction of increasing values of *C*_(vacancies)_. We did not consider other variants of grouping the samples (e.g. alloys 13, 11, 5, 7, 2 in figure 9), because in this case the alloy densities (*p* and *ρ*) would have to increase with increasing vacancy concentrations, which is unlikely.

The absence of uniform dependencies in figures 9 and 10 does not allow us to explain the decrease in *p* and *ρ* solely by an increase in the concentration of atomic vacancies. Probably, other defects or features of the physicochemical nature of the TiN_*x*_O_*y*_ alloys can also affect the *p* and *ρ* values. In addition, the disparate dependencies in figures 9 and 10 indicate the impossibility of a high correlation between the values of *C*_(vacancies)_ and *v*_l_ *,* as was observed for the values of *p* and *v*_l_ (figure 8). The corresponding dependence in figure 11 is characterized by a low *R* ^2^ < 0.200.

The graph in figure 11 shows that the highest values of *v*_l_ are characteristic of titanium oxynitrides with a vacancy concentration in the range of 2–7% (alloys numbered 8, 13, 4, 3, 11, 5, 7). However, this interval also includes the samples with low longitudinal wave velocity values. On the other hand, an increase in *C*_(vacancies)_ above 7% does not contribute to an increase in the *v*_l_ values for the corresponding samples. Thus, the vacancy concentration is an important, but not a decisive parameter in terms of finding TiN_*x*_O_*y*_ alloys with the highest *v*_l_ values.

From the above facts, it follows that there are certain dependencies between wave velocities and some physicochemical properties (*ρ*, *p* and *C*_(vacancies)_). It is possible that some correlation can be observed in the dependencies of *v*_s_ and *v*_l_ on other physicochemical parameters that are to some extent related to density. For example, with the molar mass *M*, molar volume *V* and *x*/*y* ratio in TiN_*x*_O_*y*_ alloys. In this regard, several dozen graphical dependencies between the elastic (*v*_s_, *v*_l_, *v*_m_, *G*, *B*, *σ*) and some physicochemical (*x*/*y*, *ρ*, *p*, *C*_(vacancies)_, *M*, *V*) properties of titanium oxynitrides were constructed. The basic initial data are shown in table 5 and appendix A. We tried to describe each dependence by an equation with the highest possible coefficient of determination. The results are summarized in table 7.

**Table 7.** The highest *R* ^2^ values obtained for the dependencies between the elastic and physicochemical properties of TiN_*x*_O_*y*_.

ЕP PCP	*v*_*s*_	*v*_l_	*v*_m_	*G*	*B*	*σ*
*x*/*y*	< 0.600	< 0.200	< 0.500	< 0.600	< 0.100	< 0.700
*ρ*	< 0.600	< 0.500	< 0.500	< 0.600	< 0.500	< 0.600
*р*	< 0.980	**0.988**	< 0.900	< 0.980	**0.982**	**0.984**
*С*_(vacancies)_	< 0.900	< 0.200	< 0.980	< 0.900	< 0.200	< 0.800
*M*	< 0.700	< 0.500	< 0.700	< 0.700	< 0.500	< 0.800
*V*	<0.700	< 0.100	< 0.700	< 0.600	< 0.100	< 0.700

The promising values of *R* ^2^ > 0.980 are highlighted in bold.

EP, elasticity properties; PCP, physicochemical properties.

Table 7 shows that the correlations between most of the considered elastic and physicochemical properties are low and are unlikely to find further practical application. In addition to the dependence *v*_l_ *= f*(*p*) with *R^2^* = 0.988 (figure 8), we should note good correlations between the atomic density and the values of *B* and *σ*. The corresponding equations are likely to be useful for an approximate estimate of the elastic values of the second group (*v*_l_, *B*, *σ*) for poorly studied or new titanium oxynitrides.

It is worth noting that the elastic and physicochemical properties of TiN_*x*_O_*y*_ alloys change according to a certain pattern in several directions (see figures 5 and 9). Probably, these directions can be connected with some notional cross-sections of the general Ti‒N‒O system. The identification of such connections could allow for the targeted synthesis of materials with predicted properties.

### 3.3. Interaction of components and directions of change in the elastic properties of alloys in the concentration range TiN_1.22_‒TiN_0.67_‒TiO_0.67_‒TiO_1.22_

The selected 14 TiN_*x*_O_*y*_ alloys (table 5) for the study can be considered as the products of the interaction of the initial components of the TiN_1.22_‒TiN_0.67_‒TiO_0.67_‒TiO_1.22_ concentration region (figure 12), which is part of the Ti‒N‒O ternary system (figure 1).

**Figure 13.**
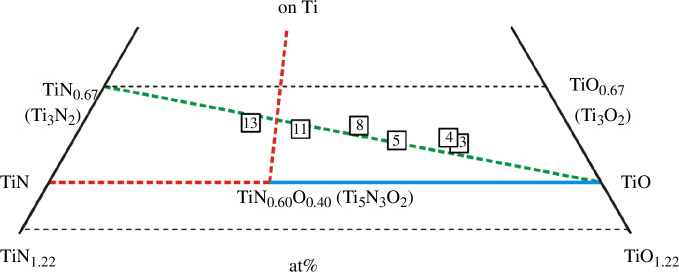
Concentration region of TiN_1.22_‒TiN_0.67_‒TiO_0.67_‒TiO_1.22_ with six TiN_*x*_O_*y*_ alloys with the highest *v*_l_ values: (i) samples numbered 3–5, 8, 11, 13 are plotted on the notional Ti_3_N_2_‒TiO cross-section, which hypothetically can exist only below 1073 K (based on the analysis of data from [81,82]); under the conditions of stabilization of alloys at 1773 K [45,49,50], this concentration region is the most promising for finding samples with the highest *v*_l_ values (table 5); (ii) the TiN‒TiO section at 1473‒1973 K consists of two cross-sections: TiN‒TiN_0.60_O_0.40_ (heterophase region) and TiN_0.60_O_0.40_‒TiO (homophase region), according to Schmitz-Dumont & Steinberg [75].

According to [45], the authors of which analysed their own results [49,50] and the data of other scientists [75,80], 16 of the 18 samples in table 1 (sources [35,36]) belong to single-phase solid solutions of the cubic structure of the NaCl type. Two more alloys, TiN_0.57_O_0.56_ (sample no. 9) and TiN_0.56_O_0.55_ (sample no. 10), are on the border between the homogeneous and heterogeneous regions in terms of phase composition (figures 1 and 12). However, most of the sources [45,49,50,75,80] are contradicted by the work of Schmitz-Dumont & Steinberg [76]. Thus, the authors of [76], when studying the TiN‒TiO section at temperatures of 1473‒1973 K, have found a wide two-phase region within the TiN‒TiN_0.6_O_0.4_ cross-section. Moreover, the TiN_0.6_O_0.4_ alloy (or Ti_5_N_3_O_2_) is a solid solution with almost identical lattice parameters to TiN [76]. If the conclusions of the authors [76] are correct, then the TiN_1.22_‒TiN_0.67_‒TiO_0.67_‒TiO_1.22_ region is subject to more complex physicochemical interactions and the homogeneity regions are somewhat different from those defined in [45]. Therefore, it cannot be ruled out that a significant part of the selected 14 alloys (table 5) will turn out to be heterophase rather than homophase. In particular, the alloys with numbers 12, 13, 14 (figure 12), of the Ti‒TiN‒Ti_5_N_3_O_2_ region (figure 13), would most likely be heterogeneous.

It should be noted that the results of [76], which were obtained almost 70 years ago, have not been confirmed in subsequent works [45,49,50,75,80]. The following experimental facts contradict the data of [76]:

— the values of density and microhardness of intermediate alloys in the Ti‒TiN‒Ti_5_N_3_O_2_ region [36,45,49];
— the crystal structures of the TiN [78,81,83], γ-TiO [79,82,84], α-Ti [84] components and the physicochemical interaction on the lateral section Ti‒N [81].

First, Schmitz-Dumont & Steinberg [76] argued the heterogeneity of alloys on the TiN‒TiN_0.6_O_0.4_(Ti_5_N_3_O_2_) segment by the constancy of their lattice parameters *a* and density *ρ*. However, according to the results of [36,45], the vast majority of the 14 TiN_*x*_O_*y*_ samples (table 5) have different values of *a* and *ρ*. Even alloys 12, 13, 14 (figure 12), which are closest to the partial section of TiN‒Ti_5_N_3_O_2_ (figure 12 and 13), are characterized by different densities (see appendix A). In addition, monograph [45] argues that the constancy of *a* and *ρ* for the alloys in the TiN‒TiN_0.6_O_0.4_ cross-section is not sufficient evidence of their heterogeneity. In particular, in [45,49], when the alloys slightly shifted from the TiN‒Ti_5_N_3_O_2_ section to the region of higher titanium content, different microhardnesses were observed and the corresponding XRD patterns showed lines of only one cubic phase (in the case of heterogeneity of the samples, their microhardness should not change, and the diffractograms should show the reflections of two different cubic phases). Similar results were also obtained by the authors of [75]: according to their X-ray studies, the alloys TiN_0.84_O_0.11_, TiN_0.72_O_0.24_ and TiN_0.64_O_0.34_ are single-phase (the compositions of these samples are shifted from the partial TiN‒TiN_0.60_O_0.40_ cross-section by only 0.5‒1.3 at% Ti).

**Figure 14.**
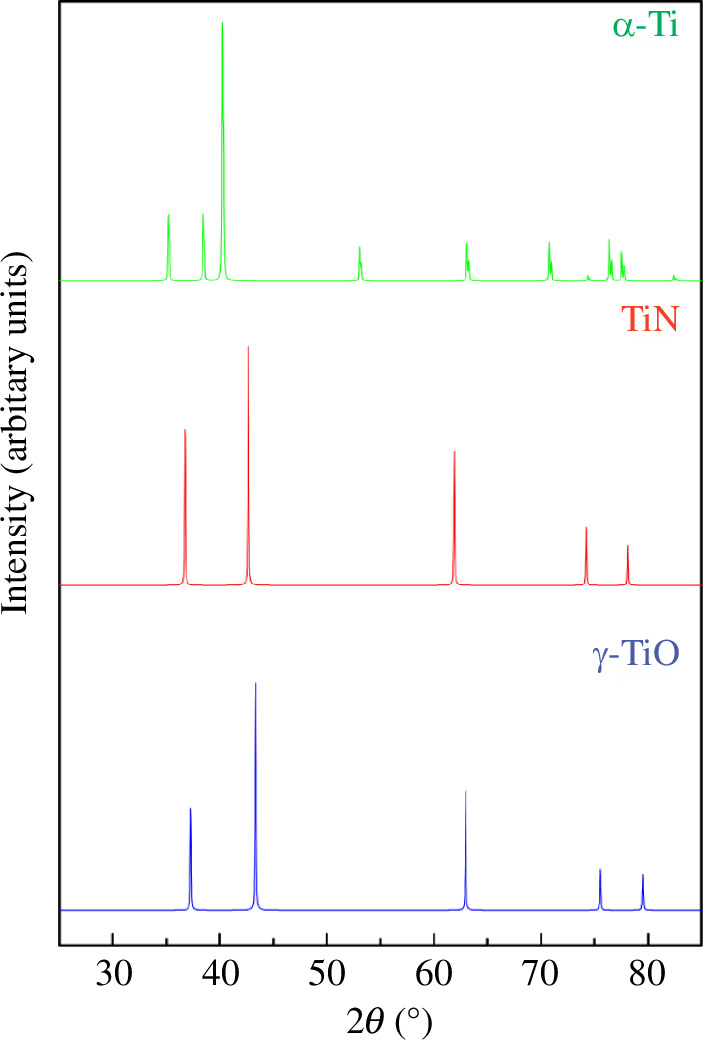
X-ray diffractograms of α-Ti [84], TiN [83], γ-TiO [84] obtained on the basis of experimental data [83,84].

Second, the results of Schmitz-Dumont & Steinberg [76] are difficult to combine with the data on the physicochemical interaction in the neighbouring Ti‒N section, namely in the partial section Ti_0.60_N_0.40_(TiN_0.67_)‒Ti_0.50_N_0.50_(TiN) [81]. Thus, at a the temperature of 1773 K (see the conditions for the synthesis of TiN_*x*_O_*y*_ alloys in §2.1), only single-phase TiN-based alloys exist in the TiN_0.67_‒TiN partial section [81], which are formed by the interaction of titanium mononitride with α-Ti. It follows that a wide region of homogeneity at the TiN_0.67_‒TiN [81] interface borders on the practically insoluble TiN‒TiN_0.60_O_0.40_ [76] region (figure 13). That is, from the comparisons of the data of [76] and [81], it is clear that TiN interacts much better with α-Ti than with γ-TiO. Here, it is advisable to compare the crystal structures of α-Ti, TiN and γ-TiO. In terms of structural parameters, TiN [83] and γ-TiO [84] are more related to each other than to α-Ti [84]. The TiN and γ-TiO compounds belong to the cubic crystal system with a lattice constant of 0.4241 nm [81] or 0.4235 nm [83] and 0.4172 nm [84], respectively. In turn, the α-titanium phase (dilute [82]) crystallizes in a hexagonal system with parameters *a = b =* 0.2944 nm, *c* = 0.4678 nm [84]. So, the significant crystal similarity between titanium nitride [83] and titanium oxide [84] and their significant difference from the α-Ti structure (figure 14) should contribute to better (even unlimited) mutual solubility of the binary components.

Although the results of Schmitz-Dumont & Steinberg [76] are difficult to agree with [81], they do not contradict the work of [82] regarding the interaction on another Ti‒O lateral section, namely in the partial section TiO_0.67_‒TiO_1.22_. In particular, the long homogeneous section of TiN_0.60_O_0.40_‒TiO [76] is well complemented by solid solutions based on the cubic phase of γ-TiO, which at 1773 K cover the concentration range of 42‒55 at% oxygen or TiO_0.72_‒TiO_1.22_ [82], or TiO_0.65_‒TiO_1.25_ [45] (see figures 1, 12 and 13).

So, based on the direct results of [45,49,50,75,80] and on the analysis of the indirect data [81,83,84], the formation of a continuous series of solid solutions (complete mutual solubility) rather than a two-phase region is more likely in the quasi-binary TiN‒TiO system at 1773 K. Therefore, the probability of the existence of a significant region of homogeneity within the TiN_1.22_‒TiN_0.67_‒TiO_0.67_‒TiO_1.22_ region (figure 12), which was reported in [45], increases. Nevertheless, one cannot unequivocally exclude the presence of some gaps in solubility and the belonging of at least several of the selected 14 samples (table 5) to heterophase alloys rather than homophase ones. It should be noted that it is easier to find some regulariies for solid solutions and to predict their properties. However, in the multi-phase samples, sharp changes of properties can sometimes be observed, which is more valuable for the search for promising materials [85].

Let us consider the change in the elastic properties of alloys within the concentration region TiN_1.22_‒TiN_0.67_‒TiO_0.67_‒TiO_1.22_. In figure 12, red arrows show 19 directions along which the longitudinal wave velocity increases in accordance with the data obtained (table 5). The green color indicates the area where the alloys with the highest *v*_l_ values are formed. Most likely, other promising titanium oxynitrides with high longitudinal wave velocity values will be located in the highlighted area.

It is worth noting that in most of the 19 directions, the values of the other parameters of the second elastic group, *B* and *σ*, also increase. Thus, the values of the bulk modulus and Poisson ratio increase in 17 of the 19 directions. The only exception is a slight decrease in the corresponding values in the two directions 2→1 and 6→9 (figure 12).

In turn, the values of the elastic properties of the first group, *v*_s_, *v*_m_ and *G*, decrease in most directions in figure 12. In particular, the shear wave velocity and shear modulus decrease in 17 out of 19 directions. The exceptions are the same directions 2→1 and 6→9, which show the opposite trend in a slight increase in the corresponding values. The average wave speed decreases in 16 directions. In addition to 2→1 and 6→9, the value of *v*_m_ increases slightly when moving from alloy no. 7 to no. 11 (figure 12).

It should be noted that the main directions in the change of properties in figure 12 are also applicable to the analysis of some physicochemical parameters of titanium oxynitrides. For example, 18 of the 19 directions in figure 12 show a decrease in the atomic density. The good agreement between the decrease in *p* values and the increase in *v_l_* values can be explained by the high degree of their mutual correlation (*R* ^2^ = 0.988 in table 7 and figure 8). At the same time, the directions of changes in other physicochemical properties that correlate less well with the longitudinal wave velocity are less obvious. In particular, the vacancy concentration, which is weakly correlated with *v*_l_ and other elastic properties (table 7 and figure 11), decreases in a directed manner only in 11 of the 19 directions shown in figure 12.

Consideration of titanium oxynitrides from the point of view of their formation in the TiN_1.22_‒TiN_0.67_‒TiO_0.67_‒TiO_1.22_ concentration region (figures 12 and 13) provides some additional advantages. In particular, the choice of directions of decrease in the atomic and conventional densities from the vacancy concentration in figures 9 and 10 coincides with certain concentration lines or notional cross-sections in the selected region (figure 12). Thus, alloys with numbers 14, 7, 3 (figure 9), 14, 12, 8, 5 (figure 9), 10, 9, 6, 1 (figures 9 and 10), 14, 12, 11, 8 (figure 10) and 14, 7, 5, 4, 3 (figure 10) can be grouped with a certain approximation within concentration sections, which mostly come from the same point but do not intersect each other. According to the principles of physicochemical analysis of complex systems used in practice [85], it is most appropriate to identify patterns in the change of alloy properties within cross-sections that do not intersect. This rejects the possibility of grouping samples according to options other than those shown in figures 9 and 10.

Establishing patterns along certain cross-sections can optimize the search for new materials with promising properties. For example, the six samples numbered 3‒5, 8, 11 and 13 with the highest *v*_l_ values (table 5) fit well on the same section of TiN_0.67_(Ti_3_N_2_)‒TiO (figure 13). The shift in the composition of these alloys from the idealized Ti_3_N_2_‒TiO section is insignificant and does not exceed ±(1‒3) at%. It is worth noting that when synthesizing samples at a temperature of 1773 K (see §2.1), the Ti_3_N_2_‒TiO cross section is most likely (figure 12) to consist of solid solutions based on cubic TiN and γ-TiO. The trigonal phase of Ti_3_N_2_ [66,86] is formed and exists at much lower temperatures of ≤1073 K [81]. This compound is probably formed by a peritectoid reaction owing to the cooling of a mixture of phases based on Ti_2_N and TiN [81]. At the same time, below 1073 K, the TiO compound exists in the form of the α-modification rather than γ-modification [82]. During slow cooling from a high temperature of 1773 K, titanium monoxide undergoes a number of polymorphic transformations. At ~1523 K, the cubic phase of γ-TiO transforms into the β-modification, and at ~(1373‒1213) K into α-modification [82]. The β-phase is a structural polytype of γ-TiO with a triple lattice constant [87], and the α-phase of TiO belongs to the monoclinic crystal system [88]. The existence of an additional phase based on TiO with a hexagonal crystal structure was also reported in [89,90]. These data [81,82,86–90] are important to consider in the low- and medium-temperature (in the temperature range from 300 K to <<1773 K) syntheses of TiN_*x*_O_*y*_ alloys. This is owing to the significant difference between the elastic properties of TiN [59–68], Ti_3_N_2_ [66,86] and Ti_2_N [66,91]. Different phases of TiO also differ significantly in terms of elastic parameters (in particular, this can be seen from the comparison of the results for cubic [40,79], hexagonal [90] and monoclinic [74,92] titanium monoxide). Probably, the medium-temperature (at 1223‒1073 K) coexistence of TiN with Ti_2_N, which at 1073 K leads to the formation of Ti_3_N_2_ [81], and various structural modifications of the initial TiO [82] can significantly affect the properties of the resulting titanium oxynitrides. It is also important to note that the literature does not contain information on the triangulation of the ternary Ti‒N‒O system at different annealing temperatures. Therefore, it is possible that at temperatures ≤1073 K, the Ti_3_N_2_‒TiO section may turn out to be non-quasi-binary, i.e. it will not meet the criteria of an independent system. Nevertheless, this particular cross-section of TiN_0.67_(Ti_3_N_2_)‒TiO at the alloy stabilization temperature of 1773 K is the most promising for finding samples with the highest longitudinal wave velocity values (see table 5 and figure 13).

Thus, the consideration of TiN_*x*_O_*y*_ alloys from the point of view of their placement on a single concentration plot (figures 12 and 13) helped to identify the main directions in the change of their elastic and physicochemical properties depending on the composition. This can be used to synthesize new titanium oxynitrides with improved elastic parameters.

### 3.4. Comparison of the elastic properties of TiN_*x*_O_*y*_ with the original components and other promising materials

The basic starting components for the synthesis of TiN_*x*_O_*y*_ intermediate alloys are TiN and TiO [49,75]. Titanium hydro-iodide and titanium dioxide can also be used to achieve the required stoichiometry [45]. Within the concentration region TiN_1.22_‒TiN_0.67_‒TiO_0.67_‒TiO_1.22_ at a stabilization temperature of 1773 K [45,49,50], only two individual compounds exist: TiN and γ-TiO (figure 12). At this temperature, the rest of the original TiN_1.22_, TiN_0.67_, TiO_0.67_, TiO_1.22_ alloys from this region are only solid solutions (TiN_0.67_ [45,81], TiO_0.67_ [45], TiO_1.22_ [45,82]) or heterogeneous mixtures (TiN_1.22_ [45], TiO_0.67_ [82]) based on or involving TiN and γ-TiO phases [45,81,82]. That is why it is advisable to compare the obtained elastic properties of the 14 samples from table 5 with the corresponding values of the original binary components TiN and γ-TiO.

It should be noted that scientific publications for TiN and γ-TiO mostly report the values of *B*, *G* and *σ* without directly indicating the values of *v*_s_ and *v*_l_. Therefore, in this work, we estimated the values of the wave velocities of these binary components using formulas (2.6)–(2.8). The density of TiN required for this purpose was taken to be 5210 kg m^−3^ [44] and for γ-TiO was 4990 kg m^−3^ [70]. As a result, it turned out that, according to various researchers, the value of *v*_l_(TiN) is in the range from 9835 to 10 882 m s^−1^ [59–68] (here, the 10 most recent available scientific articles were used), and *v*_l_(γ-TiO) is from 8045 to 8260 m s^−1^ [40,69,70]. For approximately two-thirds of the TiN_*x*_O_*y*_ samples selected for the study, the longitudinal wave velocity varies in the range of 8673‒10 960 m s^−1^ (table 5). The interval of such values can be considered commensurate or intermediate compared to the corresponding values for individual TiN [59–68] and γ-TiO [40,69,70].

It is worth noting that approximately one-third of the titanium oxynitride samples (table 5) are superior to the basic starting components TiN and γ-TiO in terms of the *v*_l_ values. This result does not contradict the traditional ideas about the directions of searching for materials with improved properties. Intermediate phases may have better elastic parameters than starting reagents. For example, the compounds TiN and γ-TiO, which are intermediate phases in the binary systems Ti‒N [81] and Ti‒O [82], have wave velocities [70,93,94] from 1.5 to more than 10 times higher than the starting α-Ti (for titanium, the estimate was based on the data from [95]), N_2_ and O_2_ (the reference data from [44] were used). In terms of some other elastic properties, TiN and γ-TiO have an advantage over the starting elemental components in the range from 2‒6 to even several thousand times (here, an approximate estimate was made based on the analysis of the data from [39,40,95–97]).

Of particular interest are the three samples, TiN_0.21_O_0.62_, TiN_0.51_O_0.27_ and TiN_0.34_O_0.45_ (table 5), which have *v*_l_ values from 18 to ~31 km s^−1^ and are ~(80‒300)% higher than that of TiN [59–68], the most efficient acoustic material among the starting components. Let us compare the results of this work with the *v*_l_ values of materials promising for acoustic resonators [72]: 18 km s^−1^ for diamond, ~13 km s^−1^ for 6H-SiC and ~11 km s^−1^ for AlN. Diamond is considered to be the material with the highest longitudinal wave velocity [43,71,72]. The question arises: can some titanium oxynitrides be on par with or even exceed diamonds in terms of longitudinal wave velocity? As §2.3 and the accuracy check in appendix A show, the probability of our calculation errors is minimized to ≤0.01%. However, only additional experiments to directly determine the longitudinal wave velocities can give an unambiguous answer.

It should be noted that the properties of materials based on TiN, TiO and TiN_*x*_O_*y*_ can significantly depend on the methods of their syntheses, technological processing, research methods, etc. [39,40,59–70,98–104]. Let us consider the influence of various factors on the elastic properties of TiN, one of the basic components for the synthesis of TiN_*x*_O_*y*_ alloys. The choice of titanium mononitride as an illustrative example is owing to the following. There are much more scientific publications on the elastic properties of TiN [39,40,59–68,78,93,94,98,99,103] than on the same properties of γ-TiO_(with vacancies)_ [40,69,70]. In turn, there is very little literature on the elastic parameters of titanium oxynitrides (see §1). The available information for TiN_*x*_O_*y*_ alloys [35–40,104] does not allow us to trace the dependence of their elastic properties on the synthesis technology or research methods. At the same time, titanium oxynitrides are sometimes considered to be products of partial oxidation of TiN [39,105], which may indicate similar dependencies in the change of their properties. Here, we will focus on considering only the minimum and maximum values of the Young’s modulus and Debye temperature for TiN (table 8), which will allow us to see the limits of the difference between different results.

**Table 8.** The minimum and maximum values of the Young’s modulus and Debye temperature selected from different sources for TiN.

source	method	*a* (nm)	*E* (GPa)	*θ*_D_ (K)
Abadias *et al*. [103]	nanoindentation	~0.424	320	524‒708 ^b^
Seifitokaldani *et al*. [59]	AIC-GGA ^a^	0.4260	429	889 ^c^ ^,^ ^f^
	AIC-LDA ^a^	0.4189	486	945 ^c^ ^,^ ^f^
Brik & Ma [63]	AIC-GGA ^a^	0.4250	429‒492	922 ^d^ ^,^ ^f^
	AIC-LDA ^a^	0.4185	479‒606	993 ^d^ ^,^ ^f^
Hu *et al*. [68]	AIC-GGA ^a^	‒	595	1083 ^c^ ^,^ ^f^
Kim *et al*. [93]	line-focus acoustic microscopy	‒	418‒556	924 ^e^ ^,^ ^f^

^a^ *Ab initio* computations with the generalized gradient approximation (AIC-GGA) or with the local density approximation (AIC-LDA).

^b^ The range of the most probable values was estimated in this work by comparing the *θ_D_*/*E* ratios for other data in this table.

^c^ Calculated here using elastic moduli from Seifitokaldani *et al*. [59] and Hu *et al*. [68].

^d^ Calculated here using intermediate bulk modulus values and constant shear modulus values from Brik & Ma [63].

^e^ Calculated here using elastic constants from Kim *et al*. [93].

^f^ In all calculations, we used formulas (2.1) [57], (2.2) [57], (2.6) [40,43], (2.7) [39,40,43,70], and the value of *ρ*(TiN) = 5210 kg m^−3^ [44].

Table 8 shows that in most of the cited works [59,63,103], the individuality of TiN was confirmed by low errors (≤2%) in the obtained lattice constant values. However, the values of the basic elastic properties (*E* and *θ_D_*) differed significantly depending on the chosen research method and sometimes on the sample preparation technology. Different approaches could lead to an almost twofold difference in the results obtained. For example, the maximum value of *E* was 606 GPa [63] and the minimum value was 320 GPa [103]. At the same time, the experimental methods of line-focus acoustic microscopy [93] and nanoindentation [103] led to differences in the obtained *E* values of 23–74%. Even within the same computational non-empirical method (AIC-GGA), the results of different studies [59,63,68] differed in the range of 13–39%. Differences in the range of 20–40% between the data obtained by different methods for TiN films, epitaxial layers and bulk samples were also reported by Abadias *et al*. [103]. It should be noted that the increase in *E* is mostly accompanied by a certain increase in *θ*_D_ (table 8). The *θ*_D_/*E* ratio ranges from 1.639 to 2.211 (as can be seen from the comparison of the data in table 8), but there is no clear correlation between the values of *θ*_D_ and *E*. Therefore, for the minimum value of the Young’s modulus of 320 GPa [103], we can approximately estimate the interval of the most probable values of *θ*_D_ = 524‒708 K. It is interesting to note that using the values of *E* = 320 GPa [103] and *θ*_D_ = 524 K (table 8) in expressions (2.1)–(2.4) leads to the value of *v*_l_(TiN) = 4.8 km s^−1^. At the same time, using the values of *E* = 320 GPa [103] and *θ*_D_ = 708 K (table 8) in (2.1)–(2.4), we obtain *v*_l_(TiN) = 13.7 km s^−1^. The first value (~4800 m s^−1^) is half as large, and the second (~13 700 m s^−1^) is 24–32% larger than the average value *v*_l_(TiN) = (9835–10 882) ≈ 10 360 m s^−1^ from the ten previously mentioned publications [59–68]. From the above, we can offer a recommendation for improving the acoustic parameters of TiN-based materials. Probably, for titanium mononitride, the highest values of *v*_l_ ~ 13.7 km s^−1^ will be observed at a ratio of *θ*_D_/*E* ~ 2.21, when *θ_D_* = 710 K and *E* ~ 320 GPa. At the same time, at *θ*_D_/*E* ~ 1.64, when *θ*_D_ approaches 520 K, the corresponding *v*_l_ values will decrease rapidly. It remains to be experimentally determined for which TiN samples (different thicknesses of amorphous or crystalline films, epitaxial layers, massive poly- or single crystals, ceramic, etc.) can achieve a close to optimal *θ*_D_/*E* ratio of 2.21. Here it is worth mentioning the results of Pang *et al*. [99], who found an almost twofold change in the value of the surface wave velocity depending on the thickness of TiN films. This confirms the significant influence of the preparation technology or synthesis features on the properties of the studied samples.

It should be noted that the elastic parameters calculated in this work for the most promising of the selected titanium oxynitrides (table 5) have a significant ‘safety margin’. The best TiN_*x*_O_*y*_ materials can remain promising even in the case of a significant decrease in their elastic characteristics owing to various modifications of the synthesis process, technological processing, or the use of other research methods. In particular, if the experiments on direct measurement of the longitudinal wave velocity, for example, for the TiN_0.34_O_0.45_ sample synthesized by different technologies, confirm the calculations (table 5) by at least 50–60% (*v*_l_ ≈ 15‒18 km  s^−1^), then this titanium oxynitride will still be able to compete with artificial diamonds [72] as an alternative material for crystal substrates of composite high-overtone bulk acoustic resonators (HBARs) [106]. Such materials have the prospect of practical application in acoustic devices for intelligent chemical and biochemical sensors [107]. In addition, these oxynitrides have additional advantages over artificial diamonds. For example, TiN_*x*_O_*y*_ samples are synthesized under low pressure at relatively low temperatures of 300 K [24], 400 K [39], 523 K [10], 1473 K [75] or 1773 K [45,49,50]. In turn, diamond production usually requires high static pressures of 13‒16 GPa, and the maximum temperature of their synthesis can reach up to 3000 K [71].

However, it should be noted here that the synthesis of titanium oxynitrides at relatively low temperatures (<1473 K) can significantly affect their physicochemical nature (capable of changing the phase composition of alloys), and thus change the values of elastic properties (probably reduce or even increase them). Fourteen selected samples (table 5) from the TiN_1.22_‒TiN_0.67_‒TiO_0.67_‒TiO_1.22_ concentration region (figure 12) during stabilizing annealing at temperatures of 1573‒1973 K [45] are likely to be single-phase solid solutions (see §3.3). Unfortunately, the literature does not contain the data on phase equilibria in the Ti‒N‒O system at temperatures lower than 1473 K. However, it is possible to approximately predict the phase transformations in this ternary system based on the phase diagrams of the initial cross-sections of Ti‒N [81] and Ti‒O [82]. It should be noted that even within the binary systems Ti‒N [81] and Ti‒O [82], the nature of the physicochemical interaction is complex and is accompanied by the formation of a number of intermediate phases, some of which undergo polymorphic transformations. Within the ternary Ti‒N‒O system, the interaction should be even more complex. Thus, at temperatures of 1213‒1523 K, all or at least some of the TiN_*x*_O_*y*_ alloys (figure 12) can transform into a three-phase mixture of β-TiO+TiN+α-Ti [81,82]. Below the temperatures of 1073‒1213 K, depending on their location in the TiN_1.22_‒TiN_0.67_‒TiO_0.67_‒TiO_1.22_ region, phases based on α-TiO, Ti_3_O_2_, TiN and Ti_3_N_2_ may appear in the structure of titanium oxynitrides [81,82] (more accurate prediction of the phase composition of alloys requires at least experimental triangulation at the above temperature intervals). It follows that the synthesis of titanium oxynitrides from the TiN_1.22_‒TiN_0.67_‒TiO_0.67_‒TiO_1.22_ region at temperatures of 300‒1473 K can contribute to their production in the form of multiphase heterogeneous mixtures. How low synthesis temperatures of the samples would affect their elastic characteristics remains unknown. This requires additional experimental studies.

As noted above, several samples of titanium oxynitrides are superior to diamonds in terms of the *v*_l_ values (see table 5 and data from [71,72]). This leads to the interest in comparing the hardness of TiN_*x*_O_*y*_ alloys and diamonds, which are best known for their unique hardness [71]. According to Vickers, the hardness *H*_V_ of diamonds is 150 GPa [71] or ~104 GPa (the value was estimated by the model of Tian *et al*. [108] using the calculated data of Fu *et al*. [109]). In turn, the titanium oxynitrides considered in this work have many times lower *H*_V_ values and are inferior to diamonds by this criterion. Among the selected 14 samples (table 5), the highest hardness of 52 GPa is demonstrated by sample no. 10 (TiN_0.56_O_0.55_) (estimated by the model [108] using data from table 5). At the same time, for the original TiN and γ-TiO components, the *H*_V_ values range from 29‒47 to 10‒15 GPa, respectively (values estimated by the model of Tian *et al*. [108] using literature data [40,59–70]). Thus, the studied titanium oxynitrides cannot compete with diamonds in terms of the *H*_V_ values. However, among TiN_*x*_O_*y*_, there are samples that are somewhat harder compared with the original TiN and γ-TiO.

**Figure 15.**
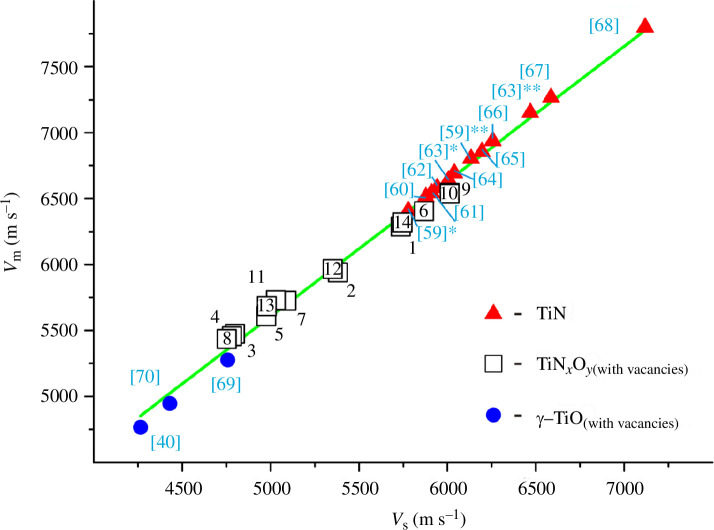
Dependence of the mean wave velocity on the shear wave velocity for TiN_*x*_O_*y*_ and the initial components TiN and γ-TiO: (i) the values for TiN (red triangles) and (ii) γ-TiO_(with vacancies)_ (blue circles) are borrowed or calculated by simple mathematical processing of data from [40,59–70] using formulas (2.2) [57], (2.6) [40,43], (2.7) [39,40,43,70] at ρ(TiN) = 5210 kg m^−3^ [44] and γ-TiO_(with vacancies)_ = 4990 kg m^−3^ [70] (notes: *GGA-method, **LDA-method [59,63]); (iii) the values for 14 TiN_*x*_O_*y*(with vacancies)_ alloys (white squares) were obtained for the first time by means of complex mathematical processing data from [35,36] (see §2 and table 5).

A comparison of the values of shear and mean wave velocities for 14 TiN_*x*_O_*y*_ alloys (table 5) with the original components TiN [59–68] and γ-TiO [40,69,70] revealed an interesting regularity (figure 15).

On the graphical dependence *v*_m_ *= f*(*v*_s_), all values fit well on the same line. A separate dependence for TiN_*x*_O_*y*_ can be described by equation (3.5) with *R^2^* = 0.996 (table 6):


(3.5) $$v_{m}\;=\;1317.492\;+\;0.868v_{s}.$$

Formula (3.5) leads to maximum calculated deviations within ±0.8%. Moreover, most of the calculated values of *v*_m_(TiN_*x*_O_*y*_) are characterized by much lower errors of ±0.2% when compared with the initial data from table 5.

The joint dependence for TiN and γ-TiO can be described by expression (3.6) with *R* ^2^ = 0.999:


(3.6) $$v_{m}\;=\;179.452\;+\;1.077v_{s}.$$

Equation (3.6) is characterized by maximum errors of up to ±0.6% for both TiN and γ-TiO. For most values of *v*_m_(TiN) from [59–68], the deviations did not exceed ± 0.1%.

A single dependence *v*_m_ *= f*(*v*_s_) for the three sets of values for TiN_*x*_O_*y*_, TiN and γ-TiO can be described by equation (3.7) with *R* ^2^ = 0.992:


(3.7) $$v_{m}\;=\;483.996\;+\;1.025v_{s}.$$

Expression (3.7) for 29 tested values (figure 15) gives maximum errors of up to ±1.9%. The smallest deviations of ± 0.6% are observed for all 12 values of *v*_m_(TiN) from [59–68].

Note that figure 15 uses data for the low-pressure phase of γ-TiO_(with vacancies)_ [40,69,70], which can contain 15–17% atomic vacancies [76,77]. A similar idealized [40] or high-pressure phase of γ-TiO_(without vacancies)_ (with 0% atomic vacancies [110,111]) is not shown in figure 15. Intermediate TiN_*x*_O_*y*_ alloys were synthesized in a vacuum [45,49,50] and also have 2–14% atomic vacancies (see §3.2).

The graph in figure 15 may have important practical implications. First, the dependence *v*_m_ *= f*(*v*_s_) is a rare example of a linear combination of values obtained using different methods. That is, equation (3.6), obtained from figure 15, almost does not depend on the method of synthesis or the method of studying the binary components (in the literature [40,59–70], γ-TiO [40,69,70] and TiN [59–68] compounds were synthesized and/or studied by both the same and different methods). Most likely, expression (3.5), like formula (3.6), will also not depend on the methods of obtaining or studying intermediate TiN_*x*_O_*y*_ alloys. Second, equation (3.7) can be used to verify or refine other calculated or experimental results. For example, for some inorganic materials it can be difficult to perform experiments to directly measure the shear wave velocity. The reason for this may be significant attenuation of ultrasound in the transverse direction, which leads to inflated measurement errors of ∼10 % [112]. Most likely, expression (3.7) will allow us to identify the most reliable *v*_s_ values among the array of experimental data, because it is not known for sure whether shear scattering of sound occurs in TiN_*x*_O_*y*_ alloys. This will also make it possible to minimize the errors in determining experimental or calculated values of *v*_s_ for TiN and γ-TiO synthesized by the latest methods, or for new or insufficiently studied intermediate TiN_*x*_O_*y*_ samples.

### 3.5. Relationship between elastic and thermal (thermodynamic and thermophysical) properties of TiN_*x*_O_*y*_

There is a close relationship between the elastic and thermal (thermodynamic and thermophysical) properties of materials. Thus, wave velocities (*v*_s_, *v*_l_, *v*_m_) can be simultaneously attributed to both elastic and thermal properties of materials [43,46,47] (see §1). In various models of thermal conductivity *λ* [47,113–117], heat transfer is directly affected by wave velocities [47,115–117] or the value of the Young’s modulus [113,114]. One of the basic parameters that allows us to relate elastic and thermal properties is the Grüneisen constant *γ*_Grüneisen_. This constant is usually determined through thermodynamic parameters [111,118–122]:


(3.8) $$\gamma_{\text{Grüneisen}}\;=\;\alpha_{V}V/\beta_{T}C_{V},$$

where *α*_V_ is the volume thermal expansion coefficient, *β*_T_ is the isothermal compressibility and *C*_V_ is the isochoric heat capacity.

Leont’ev [46] showed the possibility of calculating the thermodynamic Grüneisen constant *γ*_Güneisen_ through elastic properties:


(3.9) $$\gamma_{\text{Grüneisen}}=\alpha_{V}V/\beta_{T}C_{V}\;=\;3/2\beta_{S}\rho v_{m}^{2}=3\left(C_{11}+2C_{12}\right)/2\left(C_{11}+2C_{44}\right),$$

where *β*_S_ is the isentropic or adiabatic compressibility.

Formula (3.9) includes the values of the isochoric heat capacity *C*_V_ (thermodynamic function) and the mean wave velocity *v*_m_ (elastic or thermal parameter), which can also be combined through the Debye expression to calculate the thermophysical property, the phonon thermal conductivity *λ*_Debye_ [47]:


(3.10) $$\lambda_{Debye}\;=\;C_{V}v_{m}l/3,$$

where *l* is the average free path length of phonons.

Expression (3.10) is a mathematical record of the classical model of the phonon or minimum thermal conductivity of solids by Debye. In the more recent models of Clark [113,114] and Cahill‒Pohl [115–117], the minimum thermal conductivity depends on elastic and physicochemical properties. In particular, Clark [74,113,114] proposed the formula (3.11):


(3.11) $$\lambda_{Clarke}\;=\;0.87k_{B}E^{1/2}{\bar{M}}^{-2/3}\rho^{1/6},$$

where $\bar{M}$ is the average atomic mass.

Cahill and Pohl obtained expression (3.12) [115–117]:


(3.12) $$\lambda_{Cahill-Pohl}={\left(\pi /6\right)}^{1/3}k_{B}p^{2/3}\sum_{i}v_{i}{\left(T/\Theta_{i}\right)}^{2}\int_{0}^{\Theta_{i}/T}\frac{x^{3}\exp^{x}}{{\left(\exp^{x}-1\right)}^{2}}\;dx,$$

where *v_i_* is the sound velocity in three modes (two transverse and one longitudinal), *T* is the absolute temperature and *Θ* is the cutoff frequency for each polarization expressed.

The polarization cutoff frequency *Θ* can be calculated by equation (3.13) [115–117]:


(3.13) $$\Theta_{i}=v_{i}\left(\hbar /k_{B}\right){\left(6\pi^{2}p\right)}^{1/3},$$

where *ћ = h_P_/2π* [44].

The above equations (3.9)–(3.13) were used to calculate the thermal (thermodynamic and thermophysical) properties of titanium oxynitrides. It should be noted that formula (3.9) is valid for substances with a face-centred cubic lattice [46], which include materials of the NaCl structural type. TiN_*x*_O_*y*_ alloys also belong to this structural type [35,36,40], which allows us to use expression (3.9) for them. The transition from the wave velocities (table 5) to the elastic constants (3.9) was carried out using the known formulas (2.6)–(2.8) [39,40,43,46,70] and the density of TiN_*x*_O_*y*_ (see appendix A). The isochoric heat capacity of titanium oxynitrides (3.14) was determined from the obtained *γ*_Güneisen_ values [118,120,122]:


(3.14) $$C_{V}=C_{p}/\left(1+\alpha_{V}T\gamma_{\text{Grüneisen}}\right),$$

where *С*_р_ is the isobaric heat capacity.

It should be noted that there are currently no methods for direct experimental determination of *C*_V_ for solid materials, so indirect approaches are used through thermal expansion coefficients [47,111,122,123]. The experimental values of *α* for the TiN_*x*_O_*y*_ samples were borrowed from [35,36]. Unfortunately, the literature does not contain information on experimental studies of the isobaric heat capacity of these alloys. Therefore, in this work, the corresponding values of *C*_p_ were estimated using the Neumann‒Kopp rule [124–126]. This method has been used for various inorganic materials [127–129] and gives good results for titanium- and oxygen-containing samples [130,131]. In addition, the authors of [75] applied an approach similar to the Neumann‒Kopp rule to estimate the enthalpy of formation ‒Δ*H*_f_ of many titanium oxynitrides from the concentration region TiN_1.01_‒TiN_0.65_‒TiO_0.65_‒TiO_1.01_ (a slightly smaller region compared with TiN_1.22_‒TiN_0.67_‒TiO_0.67_‒TiO_1.22_ shown in figures 12 and 13). Their calculations and experiments were in good agreement [75]—the differences did not exceed ±(0.1‒3.3)%. It also follows from [75] that the considered TiN_*x*_O_*y*_ samples should be considered as products of the interaction of *x*TiN + *y*TiO + (1 *− x − y*)Ti (for alloys above the TiN‒TiO section, excess titanium should be taken into account). This approach minimized the calculation errors. At the same time, the use of other phases of TiN_*x*_ and TiO_*y*_ in the calculations led to significantly larger discrepancies between the experiment and the estimate [75].

It is worth noting here that the values of ‒Δ*H*_f_ are closely related to the total enthalpies *H*_T_ and *C*_p_ [123]. In particular, under standard conditions, ‒Δ*H*_f_ = *H*_T_ [123]. Sometimes the isobaric heat capacity is determined by differentiating the experimental values of the enthalpy change [132]. It follows that the errors in determining the values of ‒Δ*H*_f_ and *C*_p_ should be close.

The values of the isobaric heat capacity for titanium oxynitrides were estimated using three expressions:


(3.15) $$C_{p}(TiN_{x}O_{y})\;=\;xC_{p}(TiN)+yC_{p}(TiO)+\left\{\left(1-x-y\right)\right\}C_{p}(Ti),$$

(3.16) $$C_{p}(TiN_{x}O_{y})\;=\;xC_{p}(TiN)+(1-x)C_{p}(TiO)+\left\{(x+y-1)/2\right\}C_{p}(O_{2}),$$

(3.17) $$C_{p}(TiN_{x}O_{y})\;=\;(1-y)C_{p}(TiN)+yC_{p}(TiO)+\left\{(x+y-1)/2\right\}C_{p}(N_{2}).$$

Equation (3.15) was used to estimate the *C*_p_ for oxynitrides with an excess of titanium (alloys numbered 2‒5, 7, 8, 11‒14 in table 5). Equations (3.16) and (3.17) were used for samples with an excess of oxygen (alloys 1 and 6) and nitrogen (alloys 9 and 10), respectively. The experimental values of the isobaric heat capacity of the initial components TiN, TiO, Ti, N_2_ and O_2_ were taken from [123].

The *C*_V_ values determined by formula (3.14) were used to calculate *λ*_Debye_ according to expression (3.10). The value of *l* was estimated similarly to [41]. It was assumed that


(3.18) $$l\approx \sqrt[{3}]{V_{el.cel.}}\approx a,$$

where *V*_el. cel._ is the volume of a unit (elementary) cell.

Next, the values of *λ*_Clarke_ and *λ*_Cahill‒Pohl_ were calculated using equations (3.11)–(3.13). The values of auxiliary elastic and physicochemical parameters were taken from [35,36], table 5 and appendix A. The results obtained are grouped in table 9.

**Table 9.** Calculated thermal properties for TiN_*x*_O_*y*_ samples stabilized at 1773 K^a^.

no.	material	*γ*_Grüneisen_	*C*_p_ (J mol^−1^ K^−1^)	*C*_V_ (J mol^−1^ K^−1^)	*λ*_min_ (W m^−1^ K^−1^)		
					*λ*_Debye_	*λ*_Clarke_	*λ*_Cahill‒Pohl_
1	TiN_0.24_O_0.88_	1.054	41.022	40.628	2.733	2.190	1.620
2	TiN_0.21_O_0.75_	1.286	38.750	38.283	2.635	2.119	1.587
3	TiN_0.20_O_0.65_	3.514	37.140	35.845	2.282	2.011	1.351
4	TiN_0.21_O_0.62_	3.612	36.813	35.495	2.275	2.005	1.328
5	TiN_0.30_O_0.54_	2.312	36.703	35.894	2.412	2.048	1.508
6	TiN_0.41_O_0.70_	0.927	40.386	40.057	2.847	2.234	1.653
7	TiN_0.37_O_0.51_	2.161	37.097	36.332	2.457	2.093	1.529
8	TiN_0.34_O_0.45_	4.163	35.843	34.384	2.224	1.994	1.156
9	TiN_0.57_O_0.56_	0.847	40.577	40.290	2.995	2.299	1.688
10	TiN_0.56_O_0.55_	0.823	40.257	39.975	2.978	2.277	1.685
11	TiN_0.44_O_0.36_	3.293	35.704	34.663	2.398	2.107	1.404
12	TiN_0.52_O_0.34_	1.682	36.368	35.835	2.572	2.152	1.569
13	TiN_0.51_O_0.27_	3.734	35.204	34.034	2.337	2.086	1.307
14	TiN_0.67_O_0.23_	1.147	36.532	36.194	2.765	2.235	1.627

^a^ The conditions of heat treatment of samples are given in §2.1.

Table 9 shows that the Grüneisen constant for the selected titanium oxynitrides ranges from 0.823 to 4.163. The range of these values is somewhat wider compared to the known data for *γ*_Grüneisen_(TiN) ≈ 1.3‒4.2 (at volume changes) or ~1.9 (at constant volume) [133] and *γ*_Grüneisen_(γ-TiO_(with vacancies)_) = 1.20 [111]. For all 14 TiN_*x*_O_*y*_ samples, the traditional thermodynamic inequality *C*_p_ > *C*_V_ is fulfilled. The difference between the values of the isobaric and the isochoric heat capacities is insignificant, which is typical for solids [123].

The values of the minimum thermal conductivity obtained by different models differ slightly. The same was also observed by Tang *et al*. [74] when calculating the values of *λ*_Clarke_ and *λ*_Cahill‒Pohl_ for various titanium oxides. In this work, the following intervals of *λ*_min_ values were obtained for titanium oxynitrides: 2.224‒2.995 W m^−1^ K^−1^ (according to the Debye model), 1.994‒2.299 W m^−1^ K^−1^ (according to the Clarke model), 1.156‒1.688 W m^−1^ K^−1^ (according to the Cahill‒Pohl model). In general, within the framework of the three models used, the calculated *λ*_min_(TiN_*x*_O_*y*_) values of were in the range 1.2‒3.0 W m^−1^ K^−1^. These values are in good agreement with the known experimental data for the initial components TiN and TiO. Thus, the total thermal conductivity of TiN ranges from 1.92 to 2.09 W m^−1^ K^−1^ (data taken from the review part of [134]), and for TiO is 3.17 W m^−1^ K^−1^ (value obtained from direct experiments [135]). A slight decrease in the values of *λ*_min_(TiN_*x*_O_*y*_) compared to the initial individual components is likely owing to phonon scattering on atomic vacancies and possibly other defects in intermediate alloys. Such heat dissipation and a decrease in thermal conductivity are observed for various complex alloys [85]. Here we note that the total thermal conductivity *λ*_tot_ of materials is slightly higher than the minimum *λ*_min_ [136]:


(3.19) $$\lambda_{tot}=\lambda_{lat}+\lambda_{el}+\lambda_{phot},$$

where *λ*_lat_, *λ*_el_ and *λ*_phot_ are the lattice, electron and photon components, respectively.

A comparison of the formulas in [41] and [47] shows that *λ*_Debey_ = *λ*_phonon_ = *λ*_lat_ ≈ *λ*_min_. It is believed [41,135] that the lattice (phonon) and electronic components make the greatest contribution to the total thermal conductivity. Usually, the electronic thermal conductivity is separated from the total thermal conductivity by the Wiedemann‒Franz‒Lorenz relation [41,135,136]:


(3.20) $$\lambda_{el}=L\delta T,$$

where *L* is the Lorenz number and *δ* is the electrical conductivity.

According to the expression (3.20) in [135], it was not possible to isolate the electronic component of thermal conductivity for TiO. However, the approach from [135], in combination with the fundamental constants from [44], can be used for TiN. It turned out that *λ*_el_(TiN) is close to 0.48 W m^−1^ K^−1^ (for films with a thickness of 6 nm and an electrical conductivity of ~66 × 10^3^ S m^−1^ [101]). For TiN films with a thickness of 45 nm and a conductivity of ~2500 × 10^3^ S m^−1^ [101], the calculation according to (3.20) leads to the inequality *λ*_el_ > *λ*_tot_, which does not allow us to isolate the electronic component. It is likely that for samples with high electrical conductivity, expression (3.20) requires some correction.

Let us also estimate the values of *λ*_el_ for intermediate TiN_*x*_O_*y*_ alloys. The electrical conductivity of titanium oxynitrides strongly depends on the composition and preparation technology. These values are in a wide range: 0.004 × 10^3^ S m^−1^ [137], 0.36 × 10^3^ S m^−1^ [11], (1.4‒15.6) × 10^3^ S m^−1^ [7], 20 × 10^3^ S m^−1^ [2], (360‒3226) × 10^3^ S m^−1^ [38], 3500 × 10^3^ S m^−1^ [13]. According to formula (3.20), *λ*_el_(TiN_*x*_O_*y*_) can range from 3 × 10^−5^ W m^−1^ K^−1^ to more than 25 W m^−1^ K^−1^. Thus, the total thermal conductivity of titanium oxynitrides can either be almost the same as *λ*_min_ (table 9) or exceed it several times. It can also be noted that even the highest estimated thermal conductivity values for TiN_*x*_O_*y*_ samples (*λ*_tot_ ≈ *λ*_min_ + *λ*_el_ ≈ 2.99 + 25 ≈ 28 W m^−1^ K^−1^) are significantly lower compared to those for diamonds (~2000 W m^−1^ K^−1^ [71,138]) and some diamond composites (300‒600 W m^−1^ K^−1^ [138]).

**Figure 16.**
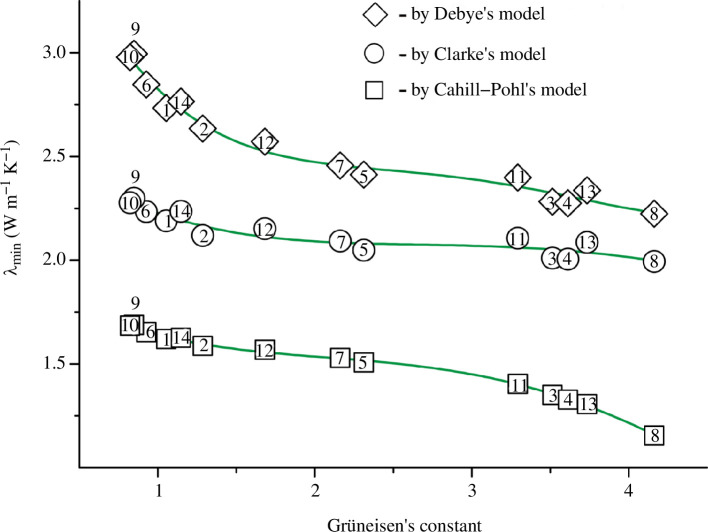
Dependence of the minimum thermal conductivity on the Grüneisen constant for TiN_*x*_O_*y*_.

The Debye, Clarke and Cahill‒Pohl models, although giving slightly different values of the minimum thermal conductivity (table 9), are characterized by similar regularities. This can be seen in the dependencies of *λ*_min_ *= f*(*γ*_Grüneisen_) in figure 16—the values of *λ*_Debye_, *λ*_Clarke_ and *λ*_Cahill‒Pohl_ decrease smoothly with increasing values of *γ*_Grüneisen_.

The course of the above curves (figure 16) is similar and is best described by polynomial equations of the fourth degree with *R* ^2^ = 0.971, 0.831 and 0.997, respectively. The highest coefficient of determination (0.997) is observed for equation (3.21):


(3.21) $$\lambda_{Cahill‒Pohl}\;=\;2.050-0.673\gamma_{Grüneisen}+0.334\gamma_{Grüneisen}^{2}-0.072\gamma_{Grüneisen}^{3}+0.004\gamma_{Grüneisen}^{4}.$$

In order to identify other promising regularities, we plotted the values of the thermal properties of titanium oxynitride similar to §3.1. The initial data are shown in table 9, and the best results of mathematical processing are collected in table 10.

**Table 10.** The highest *R^2^* values obtained during the mathematical processing of graphical dependencies between the thermal properties of the TiN_*x*_O_*y*_ alloys.

*f*(*x*)		*γ*_Grüneisen_	*C*_p_	*C*_V_	λ_Debye_	λ_Clarke_	λ_Cahill‒Pohl_
*f*(*y*)	γ_Grüneisen_	—	< 0.800	< 0.800	< 0.980	< 0.900	**0.997**
	C_p_	< 0.800	—	**0.984**	< 0.700	< 0.500	< 0.700
	C_V_	< 0.800	**0.983**	—	< 0.800	< 0.600	< 0.800
	λ_Debye_	**0.981**	< 0.700	< 0.800	—	< 0.980	**0.986**
	λ_Clarke_	< 0.900	< 0.500	< 0.600	< 0.980	—	< 0.900
	λ_Cahill‒Pohl_	**0.997**	< 0.700	< 0.800	< 0.980	< 0.900	—

The promising values of *R* ^2^ > 0.980 are highlighted in bold.

**Figure 17.**
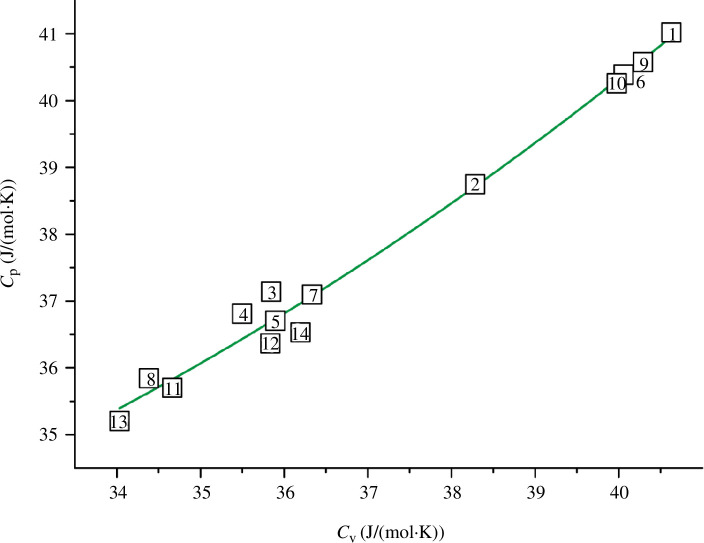
Relationship between the isobaric and isochoric heat capacities for TiN_*x*_O_*y*_.

The most promising is the dependence *C*_p_ *= f*(*C*_V_), which is shown in figure 17 and can be described by equation (3.22):


(3.22) $$C_{p}=43.344-1.135C_{V}+0.265\times 10^{-1}C_{V}^{2}.$$

Note that expression (3.22) is characterized by a not too high value *R* ^2^ = 0.984 (table 10) but leads to a slight discrepancy of ±1.22% between the calculated and initial values of *C*_p_ and *C*_V_ from table 9. It follows that equation (3.22) shows the possibility of a simplified transition from the isobaric to the isochoric heat capacity of titanium oxynitrides. At the same time, the traditional formula (3.14) and some other expressions [118,120,122] are more complicated because they require information about additional quantities: *α*_V_ (or *α*_l_—the coefficient of linear thermal expansion), *γ*_Grüneisen_, *β*_T_, *V*, etc.

**Figure 18.**
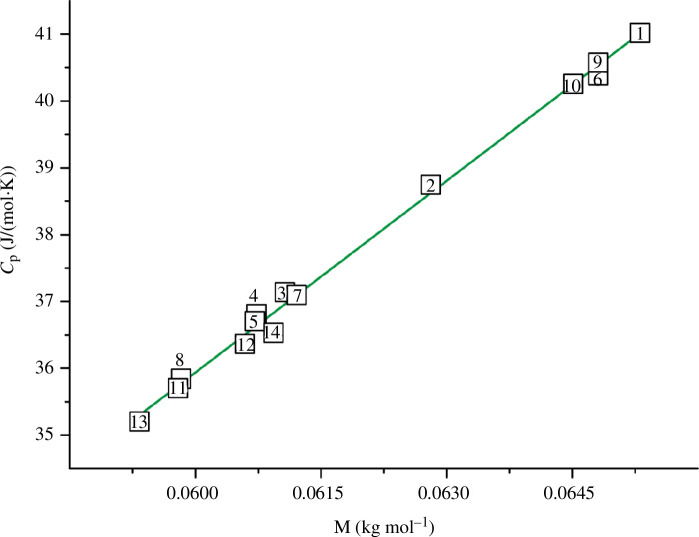
The relationship between the isobaric heat capacity and molar mass for TiN_*x*_O_*y*_.

Similarly to §3.2, we analysed the relationships between the thermal and physicochemical properties for the TiN_*x*_O_*y*_ alloys. Among the 36 graphs, the highest value of *R* ^2^ = 0.995 was observed for the dependence *C*_p_ *= f*(*M*) (table 11, figure 18, equation (3.23)):

**Table 11.** The highest *R* ^2^ values obtained during the mathematical processing of graphical dependencies between the thermal and physicochemical properties of the TiN_*x*_O_*y*_ alloys.

ТP		γ_Grüneisen_	*C*_p_	*C*_V_	λ_Debye_	λ_Clarke_	λ_Cahill‒Pohl_
PCP	*x*/*y*	< 0.700	< 0.500	< 0.600	< 0.700	< 0.600	< 0.600
	*ρ*	< 0.700	< 0.300	< 0.400	< 0.600	< 0.600	< 0.600
	*р*	**0.994**	< 0.700	< 0.800	< 0.980	< 0.900	**0.991**
	*С*_(vacancies)_	< 0.700	< 0.980	< 0.980	< 0.900	< 0.980	< 0.600
	*M*	< 0.700	**0.995**	**0.993**	< 0.700	< 0.500	< 0.600
	*V*	< 0.500	< 0.980	< 0.900	< 0.700	< 0.700	< 0.400

The promising values of *R* ^2^ > 0.980 are highlighted in bold.

PCP, physicochemical properties; TP, thermal properties.


(3.23) $$C_{p}\;=\;-21.436+956.219M.$$

Formula (3.23) leads to maximum errors within a narrow range of ±0.81%. This expression (3.23) is simpler than the three different approaches implementing the Neumann‒Kopp rule (3.15)–(3.17) that we used for the selected titanium oxynitrides and does not require data on the isobaric heat capacity of TiN, TiO, Ti, N_2_ and O_2_. It is interesting to note that equation (3.23) also gives low errors for the initial components TiN and TiO. Thus, the values of *C*_p_(TiN) = 37.729 J mol^−1^ K^−1^) and *C*_p_(TiO) = 39.634 J mol^−1^ K^−1^ calculated by equation (3.23) are close to the experimental data [123]: 37.072 J mol^−1^ K^−1^ for TiN and 39.950 J mol^−1^ K^−1^ for TiO. As can be seen, the differences are 1.77% and ‒0.79 %, respectively. Such errors are on a par with the best experimental methods. For example, differential scanning calorimetry is characterized by a discrepancy between the results within 1–2%, and that for drop calorimetry is up to 2–3% [130]. Thus, there is a reason to recommend formula (3.23) for predicting the isobaric heat capacity for many other TiN_*x*_O_*y*_ alloys.

Similar to the approaches described above, we subjected to mathematical analysis the graphical dependencies between the elastic and thermal properties of the selected titanium oxynitrides (table 12).

**Table 12.** The highest *R* ^2^ values obtained during the mathematical processing of graphical dependencies between the elastic and thermal properties of TiN_*x*_O_*y*_.

ЕP		*v*_s_	*v*_l_	*v*_m_	*G*	*B*	*σ*
ТP	γ_Grüneisen_	< 0.900	**0.987**	< 0.900	< 0.980	**0.999**	**0.999**
	*C*_p_	< 0.700	< 0.800	< 0.600	< 0.600	< 0.900	< 0.800
	*C*_V_	< 0.800	< 0.900	< 0.700	< 0.700	< 0.900	< 0.900
	λ_Debye_	**0.988**	< 0.900	< 0.980	**0.992**	< 0.980	< 0.980
	λ_Clarke_	< 0.980	< 0.800	**0.984**	< 0.980	< 0.800	< 0.900
	λ_Cahill‒Pohl_	< 0.900	< 0.980	< 0.800	< 0.900	**0.988**	**0.993**

The promising values of *R* ^2^ > 0.980 are highlighted in bold.

EP, elasticity properties; TP, thermal properties.

**Figure 19.**
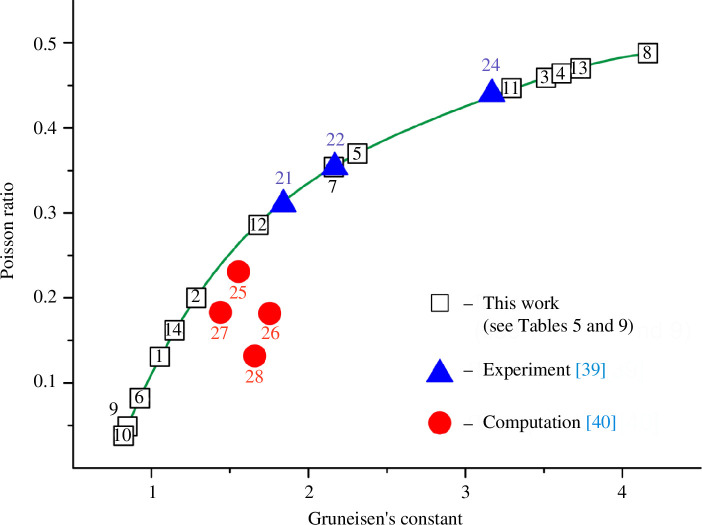
Dependence of the Poisson ratio on the Grüneisen constant for TiN_*x*_O_*y*_ alloys (numbers and composition of additional samples are detailed in figure 1).

Among the 36 graphical dependencies considered, 8 were found to have promising *R^2^* values of 0.984 – 0.999 (table 12). In particular, the dependence *σ = f*(γ_Grüneisen_), constructed for the selected 14 samples, has a high predictive potential for many other titanium oxynitrides (figure 19).

In figure 19, the values of *σ* and *γ*_Grüneisen_ for seven additional titanium oxynitrides (samples numbered 21, 22, 24–28 in figure 1) were taken from [40] or calculated using formulas (2.6)–(2.8), (3.9) by simple mathematical processing of data from [39,40]. For alloys 21, 22 and 24, the initial values were *v*_l_, *v*_s_, and *C_ij_* from [39]. At the same time, for the sample of the TiN_0.836_O_0.160_ composition [39] (no. 23 in figure 1), it was not possible to estimate the values of *σ* and *γ*_Grüneisen_ owing to the absence of its *v*_s_ and *C*_44_ values in the literature. For titanium oxynitrides 25‒28, the values of the Grüneisen constant were calculated using formula (3.9) through the elastic constants, which, in turn, were estimated by expressions (2.6)–(2.8) using the graphical values of *σ*, *B*, and *G* from [40].

Figure 19 shows that most of the points (17 out of 21) fit well on a single curve, which can be described by the following equation:


(3.24) $$\sigma =-0.514+0.953\gamma_{Grüneisen}-0.408\gamma_{Grüneisen}^{2}+0.086\gamma_{Grüneisen}^{3}-0.007\gamma_{Grüneisen}^{4}.$$

The resulting equation (3.24) has *R* ^2^ = 0.999. The maximum deviations of *σ* values for 17 samples (white squares and blue triangles in figure 19) obtained using polynomial (3.24) do not exceed ±0.004.

Note that four calculated values (4 points out of 21 in figure 19) by Xiao *et al*. [40] slightly deviate from the curve described by formula (3.24). Two alloys with numbers 25 and 27 are located close, and the other two samples (nos. 26 and 28) are farther away from the identified base curve (figure 19). This difference can be explained as follows. In [40], three idealized samples without vacancies (nos. 25, 26, 28) and one alloy (no. 27) with vacancies were considered. The extreme sample (no. 27) is close to the basic dependence, which is well fitted to alloys 1‒14, 21, 22 and 24 (figure 19), which were synthesized at low pressure [35,36,39,45] and therefore contained structural vacancies (see §3.2). In turn, alloy no. 25 (TiN_0.75_O_0.25_) falls into the concentration interval TiN_0.60_O_0.40_‒TiN(TiN_1.00_O_0.00_), which is characterized by the lowest number of vacancies ~(2‒3)% [76]. It follows that the idealized (without vacancies) sample no. 25 (TiN_0.75_O_0.25_) should differ slightly in properties from the alloy with a similar composition but with structural vacancies. This explains the proximity of sample no. 25 to the observed curve in figure 19. At the same time, alloys no. 26 (TiN_0.50_O_0.50_) and no. 28(TiN_0.25_O_0.75_) are more strongly shifted towards γ-TiO, which has a large number of structural vacancies ~(15‒17) % [76,77]. Therefore, the idealized materials, no. 26 (TiN_0.50_O_0.50_) and no. 28 (TiN_0.25_O_0.75_), should differ more in properties from alloys with structural atomic vacancies. This is confirmed by the displacement of samples 26 and 28 below the base curve in figure 19.

It can be assumed that in the case of the synthesis of the TiN_*x*_O_*y*_ alloys with numbers 1‒14, 21, 22, and 24 not under the traditional vacuum of 0.01‒0.13 Pa [45,49,50] or ~(0.2‒0.25) Pa [39], but at a high pressure of ≥7.7 GPa, which allows obtaining γ-TiO without structural vacancies [110], then atomic vacancies would probably also be filled in titanium oxynitrides. In this case, another curve would probably appear on the dependence *σ = f*(*γ*_Grüneisen_), which would pass through points nos. 26 and 28 or close to them (figure 19).

**Figure 20.**
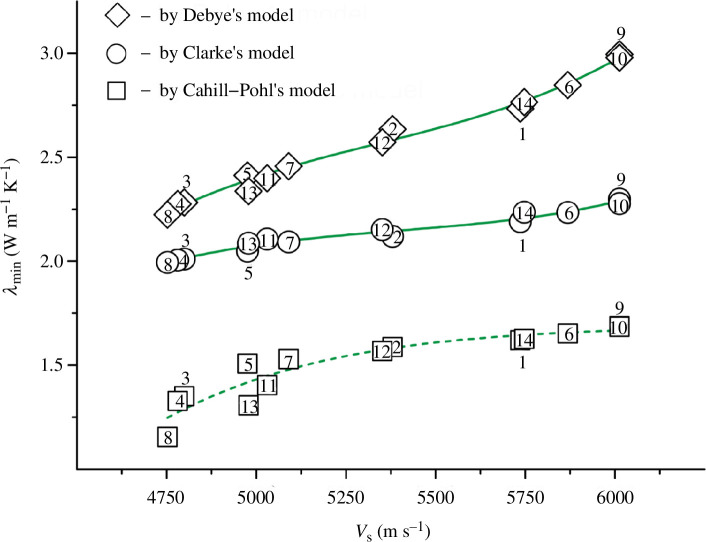
Dependence of the minimum thermal conductivity on the shear wave velocity for TiN_*x*_O_*y*_.

**Figure 21.**
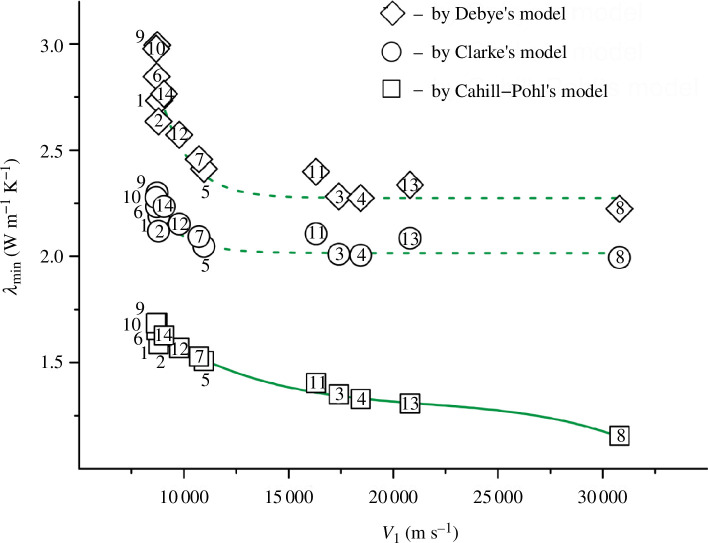
Dependence of the minimum thermal conductivity on the longitudinal wave velocity for TiN_x_O_y_.

Let us consider in more detail the dependence between wave velocities and the minimum thermal conductivity (within the three selected models), which allowed us to identify interesting regularities (figures 20 and 21).

The minimum thermal conductivity increases with the shear wave velocity (figure 20). At the same time, the values of *λ*_min_ decrease with increasing values of *v*_l_ (figure 21). It follows that the minimum thermal conductivity is proportional to the values of *v*_s_. According to Debye, the phonon or lattice thermal conductivity (3.10) is proportional to *v*_m_ (3.10) [47], which depends more strongly on the shear wave velocity. In particular, in equation (2.2) [57], 2*v*_s_ and only a single *v*_l_ are taken, and the values *v*_s_ and *v*_m_ are more strongly related to each other than to *v*_l_ (see table 6, figures 4 and 15).

According to Cahill and co-workers [115–117], the value of *λ*_min_ is also determined by a double transverse mode and only a single longitudinal mode (3.12). Thus, a decrease in the speed of sound, heat, or phonons in the transverse direction causes a decrease in the thermal conductivity of titanium oxynitrides. At the same time, even a severalfold (>300 %) increase in the *v*_l_ values cannot compensate for a 20‒27% decrease in the *v*_s_ values (when moving from sample nos. 10 to 8 in table 5), and as a result, *λ*_min_ values still decrease (table 9, figures 20 and 21).

The Clarke formula (3.11) [74,113,114] only indirectly takes into account the effect of wave velocities on the minimum thermal conductivity. The corresponding expression (3.11) includes a single elastic parameter, the Young’s modulus, which, in turn, is related to wave velocities by equation (1.2) [41,43]. At the same time, the value of *E*, and hence *λ*_Clarke_ (see equation (3.11)), is also more strongly influenced by *v*_s_ rather than *v*_l_. In particular, for titanium oxynitrides with high longitudinal and low shear wave velocities (table 5), some of the lowest values of the Young’s modulus (table 2) and the thermal conductivity *λ*_Clarke_ (table 8) are observed. And vice versa: samples with low longitudinal and high shear velocities (table 5) predominantly have high values of the Young’s modulus (table 2) and thermal conductivity *λ*_Clarke_ (table 8). However, for the selected titanium oxynitrides (tables 2 and 5), the dependencies *E = f*(*v*_s_) and *E = f*(*v*_l_) are characterized by low values of *R* ^2^ < 0.800 and *R* ^2^ < 0.500, respectively. Thus, the *λ*_Clarke_ values correlate much better with *v*_s_ and *v*_l_ (table 12 and figures 20, 21) than with the Young’s modulus.

**Figure 22.**
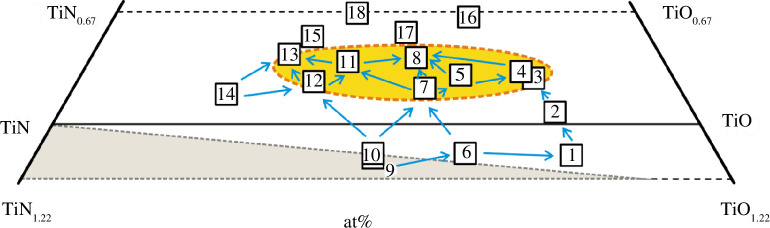
Concentration region of TiN_1.22_‒TiN_0.67_‒TiO_0.67_‒TiO_1.22_ with the main directions of decrease in the minimum thermal conductivity of the TiN_*x*_O_*y*_ alloys (according to the Debye and Cahill‒Pohl models): (i) the area of alloys with the lowest *λ*_min_ values is highlighted in yellow; (ii) the homogeneous region is highlighted in white and yellow, and the heterogeneous region at sintering temperatures of 1473‒1773 K is grey (based on the review data from [45]).

The main directions of reducing the minimum thermal conductivity of the TiN_*x*_O_*y*_ alloys within the concentration region TiN_1.22_‒TiN_0.67_‒TiO_0.67_‒TiO_1.22_ are shown in figure 22.

All 19 directions of decrease in *λ*_min_ values are well matched by the two Debye and Cahill‒Pohl models (figure 22). The Clarke model also fits well with the two models in 18 directions (the only exception is observed when moving from alloy nos. 7 to 11).

There is a significant similarity between figures 12 and 22. The increase in *v*_l_ (figure 12) in 17 of the 19 directions is consistent with a decrease in *λ*_min_ (figure 22). The notional cross-section of TiN_0.67_(Ti_3_N_2_)‒TiO (figure 13), in addition to the presence of alloys with the highest longitudinal wave velocity (see §3.3), is also the most promising for obtaining low thermally conductive titanium oxynitrides. In particular, according to the Cahill‒Pohl model, alloys from this cross-section should have a minimum thermal conductivity in the range of 1.2‒1.5 W m^−1^ K^−1^ (table 9). It follows that some TiN_*x*_O_*y*_ can be used as low-thermal conductive materials, since such samples should have values of *λ* = 1.2‒1.6 W m^−1^ K^−1^ [139–141] (for example, such materials include Ln_2_Zr_2_O_7_ [139,141] and Ti_2_InC [141]).

### 3.6. Algorithms for predicting the elastic and thermal properties of TiN_*x*_O_*y*_ through a single experimental value—the X-ray coefficient of thermal expansion or pycnometric density

The regularities identified in the previous subsections allowed us to develop two algorithms (certain sequences of applying the most effective formulas with the highest *R* ^2^ values and the lowest Δ values) for predicting the elastic and thermal properties of titanium oxynitrides based on a single experimental value. This approach is likely to significantly reduce the amount of experimental research and simplify the process of calculating unknown parameters for new TiN_*x*_O_*y*_ samples. The first of the proposed algorithms requires experimental data only on the value of thermal expansion of these alloys (table 13).

**Table 13.** The First algorithm for predicting the elastic and thermal properties of titanium oxynitrides through a single experimental value—the X-ray coefficient of thermal expansion.

algorithm step	used formula	source of the formula	error of a single formula, %	error of this algorithm, %	error of the experimental study, %
1	$C_{p}=-21.436+956.219M$	this work, equation (3.23)	± 0.81	± 0.81	≤ 3 [130,132,142]
2	$0.265\cdot 10^{-1}C_{V}^{2}-1.135C_{V}=C_{p}-43.344$	this work, equation (3.22)	± 1.22	± 0.90	unknown
3	$\gamma_{\text{Grüneisen}}=\left(C_{p}-C_{V}\right)/\alpha_{V}C_{V}T$	[118,120,122]	unknown	± (1 to >70)	unknown
4	$\sigma \;=\;-0.514+0.953\gamma_{Grüneisen}-0.408\gamma_{Grüneisen}^{2}+0.086\gamma_{Grüneisen}^{3}-0.007\gamma_{Grüneisen}^{4}$	this work, equation (3.24)	± 2.63	± (1 to >200)	unknown
5	$1/B=7.599-13.153\sigma -4.109\sigma^{2}$	this work, according to data from table 6	± 6.70	± (1 to >300)	unknown
6	$v_{l}=7497.491+7.975B-0.638\cdot 10^{-3}B^{2}$	this work, equation (3.2)	± 3.06	± (1 to >50)	~1 [48,112]
7	G=(3/2)B(1−2σ)/(1+σ)	[143–150]	± 1.79^a^	± (0‒30)	~10^b^
8	$v_{s}=2762.973+17.478G$	this work, equation (3.1)	± 2.25	± (0‒14)	~1 [48], 1‒10 [112]
9	$v_{m}=1317.492+0.868v_{s}$	this work, equation (3.5)	± 0.83	± (0‒11)	~10^b^
10	$\begin{array}{c} \lambda_{Debye}=-2.491+0.084G\\ -4.982\cdot 10^{-4}G^{2}+1.098\cdot 10^{-6}G^{3} \end{array}$	this work, according to data from table 12	± 1.84	± (0‒14)	~10^c^ [135]
11	$\begin{array}{c} \lambda_{Clarke}=-62.402+0.032v_{m}\\ -5.282\cdot 10^{-6}v_{m}^{2}+2.932\cdot 10^{-10}v_{m}^{3} \end{array}$	this work, according to data from table 12	≤ 31	± (19‒34)	~10^c^ [135]
12	$\begin{array}{c} \lambda_{Cahill-Pohl}=1.808-4.431\sigma +46.528\sigma^{2}\\ -243.299\sigma^{3}+578.196\sigma^{4}-508.599\sigma^{5} \end{array}$	this work, according to data from table 12	± 1.21	± (0‒18)	~10^c^ [135]

^a^ The error interval is determined in this work for 14 selected TiN_*x*_O_*y*_ alloys.

^b^ The expected errors for the values of *G* and *v*_m_ are given here, which strongly depend on the accuracy (90–99% [112]) of determining the value of *v*_s_ (see formulas (2.2), (2.6), §§3.1, 3.4).

^c^ The error relates to the experimental determination of the total thermal conductivity.

The independent use of each formula in table 13 is characterized by low errors (mostly 1‒3%, only for the bulk modulus ∼7% in step 5), which are even smaller than some experimental methods (1–10%). The only exceptions are the expressions in steps 3 and 11 (table 13), which require some explanation. For example, the classical equation (3.14) [118,120,122] is used in step 3, the limits of applicability of which for titanium oxynitrides remain unknown (owing to the lack of data that could be compared with each other). In addition, the formula in step 11 leads to high errors of ≤31%, although this expression has the highest *R* ^2^ = 0.984 among all the correlation equations for *λ*_Clarke_ (see table 12).

In order to determine the limits of applicability of the proposed algorithm (table 13), all expressions were checked step by step (depending on the next one from the previous one) for 14 selected TiN_*x*_O_*y*_ alloys. It turned out that this algorithm is characterized by both antagonism and synergism of calculation errors at different steps of its use. In particular, when moving from the first to the second step, the obtained *C*_V_ values were characterized by even smaller errors of ±0.90% than the independent use of the equation at the second step (±1.22% for expression (3.22) in table 13). In this case, there was an antagonism between the errors, which led to smaller errors than expected. In the third step, in addition to the calculated values of *C*_p_ and *C*_V_, we used a single experimental value for the selected titanium oxynitrides—their thermal expansion coefficients determined by the high-temperature X-ray method [35,36]. The *α*_l_ values were measured with low errors of ~3% [36]. However, small errors (1‒3%) in the values of heat capacities and thermal expansion unexpectedly led to a rapid increase in the calculated errors of *γ*_Grüneisen_ values from 1 to more than 70% (table 13). In other words, there was a synergy (mutual overlap and amplification) of errors.

The wide range of errors obtained in the third step provoked their further growth in steps 4 and 5. Thus, the calculated values of *σ* (step 4), obtained on the basis of underestimated or overestimated values of *γ*_Grüneisen_ of the third step, deviated from the expected ones in the range from 1% to more than 200%. Further, when calculating the bulk modulus, the synergy of errors increased and exceeded even 300% (step 5 in table 13). When moving from step 5 to step 6, the opposite effect was already observed—antagonism of errors. In this case, strongly underestimated or overestimated values of *B* had a weaker effect on the values of *v*_l_, which differed from the expected values within narrower ranges from 1% to >50%. Further, when calculating the values of *G, v*_s_ *, v*_m_, and *λ*_Debye_ (steps 7, 8, 9, 10), the antagonism of errors increased and even approached the permissible range for experimental errors of 1‒10%. In particular, the errors of *v*_s_ were in the range of 0‒14% (table 10). Moreover, for 13 out of 14 TiN_*x*_O_*y*_ samples, this interval was narrower than ±7.53%. The smallest errors of 0‒11% were observed for the *v*_m_ values, which for 13 alloys fluctuated within an even narrower range of ±5.93%.

Thus, the proposed First algorithm (table 13) does not allow predicting many elastic and thermal properties of titanium oxynitrides with acceptable accuracy using a single experimental parameter, the value of the coefficient of linear or volumetric thermal expansion. Nevertheless, this approach is suitable for highly accurate estimation (with expected errors of ~1 %) of individual properties, such as *C*_p_ and *C*_V_. In addition, this algorithm can be used to approximate, within ±(0‒14) %, the intervals of the most probable values of *v*_s_, *v*_m_ and *λ*_Debye_. This makes it possible to use this approach as a separate semi-empirical method or as a supplement to existing calculation methods. In particular, the estimation of the shear wave velocity values using a number of formulas in table 13 can somewhat simplify the basic calculation methodology used in this work (see §2), since for each TiN_*x*_O_*y*_ sample, it is sufficient to limit the verification of *v*_s_ values from the interval of ±14% and not to perform several hundreds or thousands of additional calculations.

The accuracy of the proposed algorithm (table 10) can be improved by using not one (*α*_l_ or *α*_V_) but several experimental or separately calculated properties of titanium oxynitrides at intermediate stages. This will make it possible to more effectively assess the elastic and thermal properties of new TiN_*x*_O_*y*_ alloys.

The Second algorithm for predicting the properties of titanium oxynitrides is based on the dependence between their longitudinal wave velocities and atomic density, which was revealed in §3.2. At the first step of this approach, a single experimental parameter is used—the pycnometric density of TiN_*x*_O_*y*_ alloys (the pycnometric method is mostly used in the study of titanium oxynitrides [45,76]). Next, the values of *v*_l_ are calculated using formulas (3.3) and (3.4). The sequence of the following calculation steps is shown in table 14.

**Table 14.** The Second algorithm for predicting the elastic and thermal properties of titanium oxynitrides through a single experimental value—pycnometric density.

algorithm step	used formula	source of the formula	error of a single formula, %	error of this algorithm, %	error of the experimental study, %
1	$p=\left(\rho /M\right)qN_{A}$	equation (3.3) and see [74]	‒	‒	‒
2	$v_{l}=8839.840$ + $\left\{51151160.160/\left(1+exp^{\left(p-8.096\right)/0.131}\right)\right\}$	this work, equation (3.4)	± 9.35	± 9.35	~1 [48,112]
3	$0.638\cdot 10^{-3}B^{2}$ −7.975B= $7497.491-v_{l}$	this work, equation (3.2)	± 3.06	± (0‒21)	unknown
4	$4.109\sigma^{2}$ +13.153σ =7.599−1/B	this work, according to data from table 6	± 6.70	± (0 ‒ >200)	unknown
5	G= (−3/2)B(2σ−1)/(1+σ)	[143–150]	± 1.79^a^	± 10.11	~10^a^
6	$v_{s}=$ 2762.973+ 17.478G	this work, equation (3.1)	± 2.25	± 4.91	~1 [48], 1‒10 [112]
7	$v_{m}=$ 1317.492 $+0.868v_{s}$	this work, equation (3.5)	± 0.83	± 4.03	~10^a^
8	$\lambda_{Debye}$ =−2.491+0.084G $-4.982\cdot 10^{-4}G^{2}$ $+1.098\cdot 10^{-6}G^{3}$	this work, according to data from table 12	± 1.84	± 7.31	~10^a^ [135]
9	$\lambda_{Cahill-Pohl}$ =1.808−4.431σ $+46.528\sigma^{2}-243.299\sigma^{3}$ $+578.196\sigma^{4}$ $-508.599\sigma^{5}$	this work, according to data from table 12	± 1.21	± 3.38	~10^a^ [135]
10	$-0.007\gamma_{Grüneisen}^{4}+0.086\gamma_{Grüneisen}^{3}$ $-0.408\gamma_{Grüneisen}^{2}+0.953\gamma_{Grüneisen}$ −0.514=σ	this work, equation (3.24)	± 2.63	± (1‒24)	unknown
11	$C_{p}=$ −21.436 +956.219M	this work, equation (3.23)	± 0.81	± 0.81	≤ 3 [130,132,142]
12	0.265⋅ $10^{-1}C_{V}^{2}$ $-1.135C_{V}$ $=C_{p}-43.344$	this work, equation (3.22)	± 1.22	± 0.90	unknown
13	$\alpha_{V}=\left(C_{p}-C_{V}\right)/\gamma_{\text{Grüneisen}}C_{V}T$	[118,120,122]	unknown	± (3 ‒ >100)	≤ 3 [35,36]

^a^ Explanation of error values is similar to table 13.

Table 11 shows that at steps 3 and 4, there is a synergy of errors, which for *σ* values exceeds 200%. However, in steps 5‒9, there is an antagonism of errors, which leads to comparable (±10%) or even smaller (3‒7%) deviations compared to direct experiments (1‒10%). Only at steps 10 and especially 13 do the calculated errors increase significantly.

The Second algorithm (table 14) has the following advantages over the First one (table 13):

— it requires only pycnometric data on the density of TiN_*x*_O_*y*_ alloys, which are much easier to obtain than the values of the thermal expansion coefficients (complex high-temperature X-ray studies are required to establish the values of *α*_l_ or *α*_V_ [35,36]);
— it allows estimating of *v*_s_ and *v*_m_ values with low errors of ≤5% (this accuracy makes it possible to compete with direct ultrasound measurements, which have Δ = ±(1‒10)% [112] (according to [43], measurement errors of sound velocity in different materials can be in the range of 0‒9%), and is better than Δ = ±(0‒14)% for the First algorithm (table 13));
— it can complement and simplify the basic computational methodology used in this paper (see §§2.2 and 2.3), as it allows for easy calculation of *v*_s_ with an accuracy of 95‒98%;
— it provides a simple way to estimate the minimum thermal conductivity according to the Debye model with errors of ±7% (according to traditional approaches, see §3.5 , this parameter *λ*_Debye_ is determined through experimental measurements of many quantities: total thermal conductivity, specific electrical conductivity, isobaric heat capacity, thermal expansion, isothermal compressibility, sound velocity, and other quantities);
— it allows predicting, with high accuracy (Δ = ±3 %), the values of the minimum thermal conductivity according to the Cahill and Pohl model (the traditional definition of the *λ*_Cahill‒Pohl_ parameter, see equation (3.12), requires information about the experimental *v*_l_ and *v*_s_ values).

It is worth noting that the proposed approaches of the Second algorithm in estimating the *λ*_Debye_ and *λ*_Cahill‒Pohl_ values are competitive owing to their narrow error ranges (1‒7% and 0‒3%, respectively). For example, one of the newest models for estimating thermal conductivity with multi-point harmonic one-dimensional convection is characterized by a slightly wider error range of 1‒11% [151]. The errors of modern thermal conductivity measurements are mostly in the range from ±(1‒3)% to ±(1‒10)% [152]. In experiments [153–155], the results for the same titanium-containing materials can differ by ~10% [154,155].

It should be noted that the Second algorithm (table 14) also has some drawbacks. In particular, it produces too high errors in the estimation of the Poisson ratio and the volumetric thermal expansion (steps 4 and 13). However, this problem also is not solved by the First algorithm (table 13), which is characterized by higher errors for a much larger number of parameters.

Thus, the Second algorithm (table 14) proved to be more successful than the First one (table 13). It allows us to predict the values of *G*, *v*_s_, *v*_m_, *λ*_Debye_, and *λ*_Cahill‒Pohl_ of titanium oxynitrides with acceptable accuracy, which in some cases is not inferior to experimental methods. The results of the verification and applicability of the Second algorithm for the TiN_*x*_O_*y*_ alloys are given in appendix B.

### 3.7. Summarizing and highlighting the most significant results

In this paper, we calculated, for the first time, the elastic properties (*v*_s_, *v*_l_, *v*_m_ *, G*, *B*, *σ*) for 14 TiN_*x*_O_*y*_ alloys that have been previously studied partially (only their *E* and *θ*_D_ values are reported in the literature [35,36,45]). The results were obtained by combining the known formulas from [39–43,46,70], modern computing capabilities [58], some auxiliary experimental data from [35,36,45], physicochemical constants [44], and stepwise long-term calculations (see §§ 2.2 and 2.3). A simplified approach was also used [41] (see expressions (1.3) and (2.5)), because the more popular equation (2.1) [51–57] and its derivative (2.4) gave, for many TiN_*x*_O_*y*_ alloys, very wide error range of 0–150% with implausible inequalities *v*_s_ > *v*_l_ and *Е <* 0 (see table 3). The use of formulas (1.3) and (2.5) allowed us to significantly reduce the range of calculated errors to 0.00–0.01% for most samples (see table 4). For 14 alloys, the obtained values of *v*_s_ and *v*_l_ were in good agreement (at the level of 99.99–100.00%) with the literature values of their *E* and *θ*_D_ (see §2.3 and appendix A). The determined values of *v*_s_ and *v*_l_ also allowed us to calculate the values of *v*_m_ *, G*, *B,* and *σ* by using formulas (2.2), (2.6)–(2.8) (see §§ 2.3, 2.4 and 3.1).

Graphical dependencies were plotted between the obtained elasticities, which allowed us to identify promising correlations (§3.1). According to the degree of correlation, the six elastic parameters were divided into two conditional groups: *v*_s_, *v*_m_, *G* (first group) and *v*_l_, *B*, *σ* (second group). High values of the coefficient of determination were observed within the respective groups: *R* ^2^ = 0.988–0.996 for the first group and *R* ^2^ = 0.984–0.999 for the second group (table 6). This allowed us to propose new correlation formulas for some elastic properties of cubic titanium oxynitrides. The most effective expressions obtained are (3.1) and (3.2) with the highest values of *R* ^2^ = 0.994 and *R* ^2^ = 0.999, respectively (figures 2 and 3, table 6). The validation of these formulas for the 14 selected TiN_*x*_O_*y*_ alloys showed high reproducibility of the results with minor calculation errors within ± 3%.

On the other hand, the cross-dependencies between the values from different groups (*v*_s_, *v*_m_, *G* with *v*_l_, *B*, *σ*) were characterized by a rather low degree of correlation, typically within *R* ^2^ = 0.500–0.960. Correlation equations with such *R* ^2^ values had insufficient predictive power and were not taken into account in this study.

Similarly, in §3.2, graphical dependencies between the six elastic parameters (*v*_s_ *, v*_l_, *v*_m_ *, G*, *B*, *σ*) and some important physicochemical properties of the selected titanium oxynitrides were plotted: *x*/*y*, *ρ*, *p*, *C*_(vacancies)_, *M* and *V*. The analysis showed that only the correlations between the atomic density (*p*) and the elastic properties of the second group (*v*_l_, *B, σ*) are promising. For such dependencies, the values of *R* ^2^ = 0.982–0.988 were observed. Equation (3.4) has the highest predictive power which *R* ^2^ = 0.988 (figure 8) and errors ranging from ±2.9% to ±(6–9)%. Other dependencies were characterized by lower *R* ^2^ values of 0.100–0.980 (table 7), and the corresponding equations led to higher errors.

In §3.3, important regularities in the changes of the elastic properties of alloys, depending on their location in the ternary Ti‒N‒O system were revealed. It was shown that the highest values of the longitudinal wave velocity are observed in the area within the concentration region TiN_1.22_‒TiN_0.67_‒TiO_0.67_‒TiO_1.22_ (figure 12). At the same time, the six TiN_*x*_O_*y*_ alloys with the maximum *v*_l_ values fit well on a single section of TiN_0.67_(Ti_3_N_2_)‒TiO (figure 13 and table 5), which is the most promising for the search for new acoustic materials.

A review of known sources [35,36,45,49,50,75,76,78,80–84] in §3.3 on the nature of the physicochemical interaction of components and phase properties in the Ti‒N‒O system has showed that the literature data are ambiguous. However, most of the results indicate the existence of an extended homogeneity area in this system, which covers almost the entire concentration range TiN_1.22_‒TiN_0.67_‒TiO_0.67_‒TiO_1.22_ (figures 1 and 12). It seems that most of the 14 studied titanium oxynitrides are solid solutions based on cubic TiN and γ-TiO. The correlations between the various properties of the selected TiN_*x*_O_*y*_ alloys identified above (§§3.1 and 3.2) can probably be explained by their single-phase nature. It is possible that the proposed correlation equations (3.1), (3.2) and (3.4), together with expressions (3.5), (3.22)–(3.24) of this work and the derivatives from tables 6 and 12, will be effecient in predicting the properties of many hundreds of other single-phase alloys from the TiN_1.22_‒TiN_0.67_‒TiO_0.67_‒TiO_1.22_ concentration region. This will allow a purposeful approach to the synthesis of TiN_*x*_O_*y*_ materials with the best properties.

In §3.4, three samples TiN_0.21_O_0.62_, TiN_0.51_O_0.27_ and TiN_0.34_O_0.45_ were selected, which have *v*_l_ values from 18 to ~31 km s^−1^ (table 5) and are able to compete with artificial diamonds for applications in acoustic resonators. From the analysis of literature data [39,40,59–70,78,93,94,98–104] (§3.4), it follows that the elastic properties of materials can vary almost twice (table 8) depending on many factors: methods of their synthesis, technological processing, research methods, etc. However, the TiN_0.34_O_0.45_ alloy, as the most promising sample identified in this work, has a ‘high margin of technological strength’. In §§2.1 and 3.3, it is stated that to obtain single-phase titanium oxynitrides, high-temperature synthesis at ≥1773 K should be used [45,49,50]. However, in modern works, much lower temperatures of ≤523 K are used [10,24,39]. Most likely, low-temperature syntheses of titanium oxynitrides will promote the formation of multiphase mixtures rather than solid solutions. This is likely to contribute to either deterioration or even improvement of the elastic properties of the respective alloys (there are no experimental data for TiN_*x*_O_*y*_ in this regard). Nevertheless, even with a significant negative effect—a twofold decrease in the longitudinal wave velocity (*v*_l_ ~ 15–16 km s^−1^)—TiN_0.34_O_0.45_ will continue to compete with artificial diamonds owing to the energy gains from simplifying the synthesis conditions. If the negative effect turns out to be even greater and leads to a threefold decrease in the corresponding value (*v*_l_ ~ 10–11 km s^−1^), the sample of TiN_0.34_O_0.45_ composition will still not lose its prospects, since it will be able to compete with other acoustic materials for micromechanical resonators (e.g. Si, AlN, etc., which have *v*_l_ ~ 9–11 km s^−1^ [72]).

Since some titanium oxynitrides are able to compete with diamonds in terms of acoustic properties, it was interesting to compare the Vickers *H*_V_ values for these materials (since diamonds have record values of *H*_V_ = 150 GPa [71]). It turned out that the 14 selected TiN_*x*_O_*y*_ alloys are much inferior to artificial diamonds in this parameter and the highest hardness of ~52 GPa is expected for the TiN_0.56_O_0.55_ sample (estimates were made according to the model [108] using the calculated elastic properties from table 5).

It was noted above that the elastic properties of materials can be poorly reproduced if they are synthesized or studied by different methods (see table 8). At the same time, in this work, a rare dependence was found on the graph *v*_m_ *= f*(*v*_s_) (figure 15), which directly combines the values obtained by different methods. A separate dependence for TiN_*x*_O_*y*_ has *R* ^2^ = 0.996 (table 6) and can be described by equation (3.5), and the general dependence for TiN_*x*_O_*y*_, TiN and γ-TiO can be described by expression (3.7) with *R* ^2^ = 0.992. Formula (3.5) gives maximum calculated deviations within ±0.8%, and equation (3.7) up to ±1.9%. These expressions, (3.5) and (3.7), can also be used to verify new experimental or calculated values of *v*_s_ and *v*_m_ for TiN, TiN_*x*_O_*y*(with vacancies)_, and γ-TiO_(with vacancies)_.

In §3.5, the values of a number of thermal (thermodynamic and thermophysical) properties were calculated for the first time for 14 selected titanium oxynitrides: *γ*_Grüneisen_, *C*_p_, *C*_V_, *λ*_min_ by Debye᾽s model, *λ*_min_ by Clark᾽s model, *λ*_min_ by Cahill‒Pohl’s model (see table 9). The values of the Grüneisen constant and the minimum thermal conductivity calculated for 14 TiN_*x*_O_y_ alloys by the three different models were intermediate or slightly lower compared to the literature data for the original TiN [133,134] and TiO [111,135] components. Similarly to §3.1, graphs were drawn between the obtained values of the thermal properties of TiN_*x*_O_*y*_. The most promising one is the dependence *C*_p_ *= f*(*C*_V_) (figure 17), which can be described by equation (3.22). Expression (3.22) is characterized by a not very high value of *R* ^2^ = 0.984 (table 10), but leads to small calculation errors of ±1.2%. Equation (3.22) makes it easy to switch from isobaric to isochoric heat capacity, unlike the classical formulas from [118,120,122], which require many auxiliary quantities such as: *α*_V_ or *α*_l_, γ_Grüneisen_, *β*_T_, *V*, etc.

Similarly to §3.2, we analysed the correlations between the thermal and physicochemical properties of titanium oxynitrides (table 11). The highest value of *R* ^2^ = 0.995 was observed for the relationship *C*_p_ *= f*(*M*). The corresponding equation (3.23) resulted in insignificant errors of ±0.8 %, which are lower compared to the best experimental methods (from 1–2% to 2–3% [130]). For TiN_*x*_O_*y*_ samples, formula (3.23) can also compete with the well-known empirical Neumann‒Kopp rule, since it is simple and does not require data on the isobaric heat capacity of the starting materials TiN, TiO, Ti, N_2_ and O_2_.

Among the cross-dependencies between the thermal and elastic properties of TiN_*x*_O_*y*_ alloys (table 12), it is worth noting the graph *σ = f*(*γ*_Grüneisen_) in figure 19. The observed curve is well described by equation (3.24), which has *R* ^2^ = 0.999 and allows calculating *σ* values with a high accuracy of ± 0.004.

Within the concentration region TiN_1.22_‒TiN_0.67_‒TiO_0.67_‒TiO_1.22_, the values of *λ*_min_ calculated by the three models decrease (figure 22) mainly in the directions of increasing the corresponding values of *v*_l_ (figure 12) and decreasing the values of *v*_s_ and *v*_m_ (see §3.3). The notional cross-section of TiN_0.67_(Ti_3_N_2_)‒TiO (figure 13), in addition to the presence of alloys with the highest longitudinal wave velocity (see §3.3), is also the most promising for obtaining low thermally conductive titanium oxynitrides. According to the Cahill‒Pohl model, the corresponding samples have a minimum thermal conductivity in the range of ∼1.2–1.5 W m^−1^ K^−1^ (nos. 3–5, 8, 11, 13 in table 9). If their total (experimental) thermal conductivity is close to the minimum, they will be able to compete with modern thermal insulators Ln_2_Zr_2_O_7_ [139,141] and Ti_2_InC [141].

The most efficient equations (3.1)–(3.5) and (3.22)–(3.24), some derivatives of expressions from tables 6 and 12, and three well-known formulas from [74,118,120,122,143–150] (see tables 13 and 14) were used to develop two algorithms for predicting the properties of TiN_*x*_O_*y*_ through a single experimental parameter—the X-ray coefficient of thermal expansion (the First algorithm) or the pycnometric density (the Second algorithm) (see §3.6). Such algorithms allow to minimize the amount of experimental researches (in fact, reducing it to the determination of a single parameter) and to significantly simplify the process of calculating unknown properties for new titanium oxynitrides.

The lowest errors were demonstrated by the Second algorithm, which requires information only on the pycnometric density of TiN_*x*_O_*y*_ alloys. It allows:

1) to determine the isobaric and isochoric heat capacities with errors of ±0.8 and ±0.9%, respectively;

2) to estimate the values of *v*_s_ and *v*_m_ with errors ≤ 5%;

3) to simplify and speed up the basic computational methodology used in this work (see §§2.2 and 2.3), since it allows us to quickly calculate *v*_s_ with an accuracy of 95–98%;

4) to estimate the minimum thermal conductivity within the framework of the Debye model with errors of ±7%;

5) to calculate the values of the minimum thermal conductivity within the framework of the Cahill‒Pohl model with errors of ±3%.

The proposed algorithm can be used to predict, with an acceptable accuracy, some elastic, thermodynamic and thermophysical values for many hundreds of new, mostly single-phase, titanium oxynitrides.

## 4. Conclusions

For the first time, the values of a number of elastic properties were obtained for 14 selected titanium oxynitrides by the calculation semi-empirical method: the shear *v*_s_, the longitudinal *v*_l_, and the mean *v*_m_ wave velocities, the shear modulus *G*, the bulk modulus *B* and the Poisson ratio *σ*. According to the high indices of mutual correlation, these parameters are grouped into two elastic groups: the first group (*v*_s_ *, v*_m_ *, G*) and the second one (*v*_l_ *, B, σ*). It has been found that the graphical dependencies of elastic values within a particular group are characterized by reasonably high coefficients of determination *R* ^2^ = 0.984‒0.999. However, the values from different elastic groups correlate with each other much worse (*R* ^2^ = 0.500‒0.960).

It has been found that the elastic parameters of the selected TiN_*x*_O_*y*_ samples correlate weakly with their physicochemical properties: the *x*/*y* ratio in the composition of alloys, the experimental density *ρ*, the concentration of atomic vacancies *C*_(vacancies)_, the molar mass *M* and the molar volume *V* (*R* ^2^ = 0.100‒0.980). At the same time, the best correlation (*R* ^2^ = 0.982‒0.988) was observed between the elastic parameters of the second group (*v*_l_ *, B, σ*) and the atomic density *p*, which is calculated on the basis of the pycnometric density *ρ*.

The analysis of the known data on the physicochemical interaction of components in the TiN_1.22_‒TiN_0.67_‒TiO_0.67_‒TiO_1.22_ concentration region and the calculated elastic properties allowed us to identify the main directions in the change in the values of *v*_l_ *, B, σ, v*_s_ *, v*_m_ *, G* depending on the composition of TiN_*x*_O_*y*_ alloys. It was found that the highest values of longitudinal and the lowest values of shear wave velocities were observed for the samples from the TiN_0.67_(Ti_3_N_2_)‒TiO section. Alloys of this concentration line, provided they are stabilized at 1773 K, are most likely solid solutions based on cubic TiN and γ-TiO. At this temperature, the TiN_0.67_(Ti_3_N_2_)‒TiO cross-section is the most promising for the search for new titanium oxynitrides with improved acoustic and elastic characteristics.

It has been found that most of the selected titanium oxynitrides have intermediate wave velocities compared to the basic starting components TiN and γ-TiO. At the same time, some samples of TiN_0.34_O_0.45_, TiN_0.51_O_0.27_, TiN_0.21_O_0.62_ with *v*_l_ = 18‒31 km s^−1^ are able to compete with traditional materials for acoustic resonators, in particular, artificial diamonds. Based on the analysis of various data for TiN as a precursor of TiN_*x*_O_*y*_, it is assumed that the use of different methods of research, synthesis, or technological processing should not significantly reduce the competitiveness and practical prospects of these titanium oxynitrides. It was found that the joint graph of *v*_m_ *= f*(*v*_s_) for TiN, γ-TiO_(with vacancies)_, and TiN_*x*_O_*y*(with vacancies)_ shows a rare linear dependence with a high *R* ^2^ = 0.992, which is not affected by the methods of synthesis and studying these materials.

The determined elastic parameters in combination with known empirical and semi-empirical formulas allowed us to calculate the thermal (thermodynamic and thermophysical) properties of 14 selected titanium oxynitrides: the Grüneisen constant *γ*_Grüneisen_, the isobaric *C*_p_ and the isochoric *C*_V_ heat capacities, the minimum thermal conductivity according to the Debye *λ*_Debye_, the Clarke *λ*_Clarke_, the Cahill and Pohl *λ*_Cahill‒Pohl_. The calculated values of *λ*_min_(TiN_*x*_O_*y*_) = 1.2‒3.0 W m^−1^ K^−1^ obtained from the three models are intermediate or slightly lower than the literature experimental data for the total thermal conductivity of the initial components TiN and TiO. Among more than a hundred analysed graphical dependencies between the thermal, physicochemical and elastic properties of the TiN_*x*_O_*y*_ samples, only 12 were found to have promising values of the coefficient of determination *R* ^2^ = 0.981‒0.999. The dependencies of *λ*_Debye_, *λ*_Clarke_ and *λ*_Cahill‒Pohl_ on the elastic properties of titanium oxynitrides are characterized by similar regularities: the minimum thermal conductivity increases with *v*_s_ and decreases with increasing *v*_l_. The TiN_0.67_(Ti_3_N_2_)‒TiO section, in addition to alloys with the highest longitudinal wave velocity, is also the most promising for the search for new low-thermal-conductivity titanium oxynitrides. According to the Cahill‒Pohl model, samples of this concentration band should have *λ*_min_ = 1.2‒1.5 W m^−1^ K^−1^, which meets the current criteria for promising low-thermal-conductivity materials.

Two algorithms (semi-empirical methods) have been developed to predict the properties of TiN_*x*_O_*y*_ alloys based on a single experimental parameter, the X-ray coefficient of thermal expansion (the First algorithm) or pycnometric density (the Second algorithm). The proposed algorithms consist of the step-by-step use of the identified relationships with the highest *R* ^2^ values and the lowest Δ errors in combination with some literature formulas. It turned out that the semi-empirical method based on the experimental density has the highest accuracy, which allows estimating the values of the shear *v*_s_ and the mean *v*_m_ wave velocities (Δ= ±(1‒5)%), the minimum thermal conductivity *λ*_min_ within the framework of the Cahill‒Pohl model (Δ= ±(0‒3)%), the isobaric *C*_p_ and the isochoric *C*_V_ heat capacities (Δ < 1%) with low errors. Known experimental methods and alternative models for determining these quantities are characterized by wider error intervals. The developed algorithm based on the pycnometric density can be used to predict, with an acceptable accuracy, some elastic, thermodynamic, and thermophysical quantities for many hundreds of new, mostly single-phase, titanium oxynitrides.

The approaches proposed in this paper to identify materials with the most promising elastic and thermal properties can be used for numerous similar samples of oxynitrides, oxycarbides, carbonitrides, carbides, nitrides, oxides of titanium, zirconium, hafnium, vanadium, niobium, tantalum and others.

## Data Availability

All the data that confirm the results of the present work and could be used to verify these results are presented in the main text of the article and appendices A and B.

## Appendix A

Verification of the applicability of the calculated *v*_s_ and *v*_l_ values in order to achieve the optimal agreement between *E* and *θ*_D_.

Basic information:

1. for verification, we used the formulas (1.2) and (1.3) from sources [41–43];
2. the values of the required constants were taken from [44]: *h*_P_ = 6.62606876 × 10^−34^ J s; *k*_B_ = 1.3806503 × 10^−23^ J K^−1^; *N*_A_ = 6.02214199 × 10^23^ mol^−1^; *π* = 3.14159265;
3. the values of *E* and *θ*_D_ for 18 TiN_*x*_O_*y*_ alloys were taken from [35,36] (see table 1) and were considered ‘true’;
4. the values of *v*_s_ and *v*_l_ were calculated in this work (see table 5) relative to the ‘true’ values of Young’s modulus and Debye temperature;
5. the values of *M* and *ρ* for TiN_*x*_O_*y*_ alloys are given in table 15.

**Table 15.** Molecular weight and density of selected 18 titanium oxynitrides.

sample	*M* (kg mol^−1^)^a^	*ρ* (kg m^−3^)^b^	sample	*M* (kg mol^−1^)^a^	*ρ* (kg m^−3^)^b^	sample	*M* (kg mol^−1^)^a^	*ρ* (kg m^−3^)^b^
TiN_0.24_O_0.88_	0.06530808	4970	TiN_0.37_O_0.51_	0.06120917	5130	TiN_0.51_O_0.27_	0.05933026	5080
TiN_0.21_O_0.75_	0.06280796	5180 ^еst^	TiN_0.34_O_0.45_	0.05982901	5060	TiN_0.67_O_0.23_	0.06093135	5210
TiN_0.20_O_0.65_	0.06106795	5060	TiN_0.57_O_0.56_	0.06481048	5210	TiN_0.45_O_0.28_	0.05864985	5080
TiN_0.21_O_0.62_	0.06072804	5080	TiN_0.56_O_0.55_	0.06451042	5200	TiN_0.22_O_0.46_	0.05830820	5100
TiN_0.30_O_0.54_	0.06070869	5160 ^еst^	TiN_0.44_O_0.36_	0.05978973	5120	TiN_0.32_O_0.40_	0.05874890	5080
TiN_0.41_O_0.70_	0.06480933	5100	TiN_0.52_O_0.34_	0.06059028	5160	TiN_0.36_O_0.31_	0.05786923	5000

^a^ Calculated from the atomic weights of titanium, nitrogen and oxygen given in [44].

^b^ The *ρ* values for 16 TiN_*x*_O_*y*_ samples were taken from [36]; for TiN_0.21_O_0.75_ and TiN_0.30_O_0.54_ alloys, the densities were estimated ^(est)^ in this work.

The values of *θ*_D_, *E* and the errors in their calculations obtained using the *v*_s_ and *v*_l_ values proposed in this work:

$E\left(TiN_{0.24}O_{0.88}\right)$ $=4970\cdot 5736^{2}\left(3\cdot 8805^{2}-4\cdot 5736^{2}\right)$ $/\left(8805^{2}-5736^{2}\right)=370.00$ (error of *E* is 0.00 GPa or 0.00%),

$\theta_{D}\left(TiN_{0.24}O_{0.88}\right)$ $=479.92375477\cdot 10^{-13}\times$ ${\left(54.19927791\cdot 10^{23}/\left(165.12787765\cdot 10^{-6}\times \left(2\cdot 5736^{-3}+8805^{-3}\right)\right)\right)}^{0.33333333}=670.02$ (error of *θ_D_* is 0.02 K or 0.00%);

$E\left(TiN_{0.21}O_{0.75}\right)$ $=5180\cdot 5380^{2}\left(3\cdot 8790^{2}-4\cdot 5380^{2}\right)$ $/\left(8790^{2}-5380^{2}\right)=359.98$ (error of *E* is 0.02 GPa or 0.00%),

$\theta_{D}\left(TiN_{0.21}O_{0.75}\right)$ $=479.92375477\cdot 10^{-13}\times$ ${\left(54.19927791\cdot 10^{23}/\left(152.36836190\cdot 10^{-6}\times \left(2\cdot 5380^{-3}+8790^{-3}\right)\right)\right)}^{0.33333333}=650.03$ (error of *θ_D_* is 0.03 K or 0.00%);

$E\left(TiN_{0.20}O_{0.65}\right)$ $=5060\cdot 4799^{2}\left(3\cdot 17394^{2}-4\cdot 4799^{2}\right)$ $/\left(17394^{2}-4799^{2}\right)=340.00$ (error of *E* is 0.00 GPa or 0.00%),

$\theta_{D}\left(TiN_{0.20}O_{0.65}\right)$ $=479.92375477\cdot 10^{-13}$ $\times {\left(54.19927791\cdot 10^{23}/\left(151.66057144\cdot 10^{-6}\times \left(2\cdot 4799^{-3}+17394^{-3}\right)\right)\right)}^{0.33333333}=600.03$ (error of *θ_D_* is 0.03 K or 0.01%);

$E\left(TiN_{0.21}O_{0.62}\right)$ $=5080\cdot 4781^{2}\left(3\cdot 18433^{2}-4\cdot 4781^{2}\right)$ $/\left(18433^{2}-4781^{2}\right)=339.98$ (error of *E* is 0.02 GPa or −0.01%),

$\theta_{D}\left(TiN_{0.21}O_{0.62}\right)$ $=479.92375477\cdot 10^{-13}$ $\times {\left(54.19927791\cdot 10^{23}/\left(150.22264891\cdot 10^{-6}\times \left(2\cdot 4781^{-3}+18433^{-3}\right)\right)\right)}^{0.33333333}=600.03$ (error of *θ_D_* is 0.03 K or 0.01%);

$E\left(TiN_{0.30}O_{0.54}\right)$ $=5160\cdot 4975^{2}\left(3\cdot 10960^{2}-4\cdot 4975^{2}\right)$ $/\left(10960^{2}-4975^{2}\right)=350.00$ (error of *E* is 0.00 GPa or 0.00%),

$\theta_{D}\left(TiN_{0.30}O_{0.54}\right)$ $=479.92375477\cdot 10^{-13}$ $\times {\left(54.19927791\cdot 10^{23}/\left(147.84649170\cdot 10^{-6}\times \left(2\cdot 4975^{-3}+10960^{-3}\right)\right)\right)}^{0.33333333}=620.01$ (error of *θ_D_* is 0.01 K or 0.00%);

$E\left(TiN_{0.41}O_{0.70}\right)$ $=5100\cdot 5868^{2}\left(3\cdot 8696^{2}-4\cdot 5868^{2}\right)$ $/\left(8696^{2}-5868^{2}\right)=380.02$ (error of *E* is 0.02 GPa or 0.00%),

$\theta_{D}\left(TiN_{0.41}O_{0.70}\right)$ $=479.92375477\cdot 10^{-13}$ $\times {\left(54.19927791\cdot 10^{23}/\left(159.68981551\cdot 10^{-6}\times \left(2\cdot 5868^{-3}+8696^{-3}\right)\right)\right)}^{0.33333333}=690.03$ (error of *θ_D_* is 0.03 K or 0.00%);

$E\left(TiN_{0.37}O_{0.51}\right)$ $=5130\cdot 5090^{2}\left(3\cdot 10715^{2}-4\cdot 5090^{2}\right)$ $/\left(10715^{2}-5090^{2}\right)=359.99$ (error of *E* is 0.01 GPa or 0.00%),

$\theta_{D}\left(TiN_{0.37}O_{0.51}\right)$ $=479.92375477\cdot 10^{-13}$ $\times {\left(54.19927791\cdot 10^{23}/\left(149.93705950\cdot 10^{-6}\times \left(2\cdot 5090^{-3}+10715^{-3}\right)\right)\right)}^{0.33333333}=630.01$ (error of *θ_D_* is 0.01 K or 0.00%);

$E\left(TiN_{0.34}O_{0.45}\right)$ $=5060\cdot 4752^{2}\left(3\cdot 30795^{2}-4\cdot 4752^{2}\right)$ $/\left(30795^{2}-4752^{2}\right)=340.00$ (error of *E* is 0.00 GPa or 0.00%),

$\theta_{D}\left(TiN_{0.34}O_{0.45}\right)$ $=479.92375477\cdot 10^{-13}$ $\times {\left(54.19927791\cdot 10^{23}/\left(148.58369311\cdot 10^{-6}\times \left(2\cdot 4752^{-3}+30795^{-3}\right)\right)\right)}^{0.33333333}=599.95$ (error of *θ_D_* is 0.05 K or −0.01%);

$E\left(TiN_{0.57}O_{0.56}\right)$ $=5210\cdot 6012^{2}\left(3\cdot 8729^{2}-4\cdot 6012^{2}\right)$ $/\left(8729^{2}-6012^{2}\right)=394.99$ (error of *E* is 0.01 GPa or 0.00%),

$\theta_{D}\left(TiN_{0.57}O_{0.56}\right)$ $=479.92375477\cdot 10^{-13}$ $\times {\left(54.19927791\cdot 10^{23}/\left(156.32101928\cdot 10^{-6}\times \left(2\cdot 6012^{-3}+8729^{-3}\right)\right)\right)}^{0.33333333}=710.02$ (error of *θ_D_* is 0.02 K or 0.00%);

$E\left(TiN_{0.56}O_{0.55}\right)$ $=5200\cdot 6012^{2}\left(3\cdot 8673^{2}-4\cdot 6012^{2}\right)$ $/\left(8673^{2}-6012^{2}\right)=390.00$ (error of *E* is 0.00 GPa or 0.00%),

$\theta_{D}\left(TiN_{0.56}O_{0.55}\right)$ $=479.92375477\cdot 10^{-13}$ $\times {\left(54.19927791\cdot 10^{23}/\left(155.89650871\cdot 10^{-6}\times \left(2\cdot 6012^{-3}+8673^{-3}\right)\right)\right)}^{0.33333333}=710.01$ (error of *θ_D_* is 0.01 K or 0.00%);

$E\left(TiN_{0.44}O_{0.36}\right)$ $=5120\cdot 5030^{2}\left(3\cdot 16307^{2}-4\cdot 5030^{2}\right)$ $/\left(16307^{2}-5030^{2}\right)=375.00$ (error of *E* is 0.00 GPa or 0.00%),

$\theta_{D}\left(TiN_{0.44}O_{0.36}\right)$ $=479.92375477\cdot 10^{-13}$ $\times {\left(54.19927791\cdot 10^{23}/\left(146.74607524\cdot 10^{-6}\times \left(2\cdot 5030^{-3}+16307^{-3}\right)\right)\right)}^{0.33333333}=634.99$ (error of *θ_D_* is 0.01 K or 0.00%);

$E\left(TiN_{0.52}O_{0.34}\right)$ $=5160\cdot 5351^{2}\left(3\cdot 9774^{2}-4\cdot 5351^{2}\right)$ $/\left(9774^{2}-5351^{2}\right)=380.00$ (error of *E* is 0.00 GPa or 0.00%),

$\theta_{D}\left(TiN_{0.52}O_{0.34}\right)$ $=479.92375477\cdot 10^{-13}$

$\times {\left(54.19927791\cdot 10^{23}/\left(147.55812272\cdot 10^{-6}\times \left(2\cdot 5351^{-3}+9774^{-3}\right)\right)\right)}^{0.33333333}=659.97$ (error of *θ*_*D*_ is 0.03 K or 0.00%);

$E\left(TiN_{0.51}O_{0.27}\right)$ $=5080\cdot 4978^{2}\left(3\cdot 20792^{2}-4\cdot 4978^{2}\right)$ $/\left(20792^{2}-4978^{2}\right)=370.00$ (error of *E* is 0.00 GPa or 0.00%),

$\theta_{D}\left(TiN_{0.51}O_{0.27}\right)$ $=479.92375477\cdot 10^{-13}$ $\times {\left(54.19927791\cdot 10^{23}/\left(146.76496702\cdot 10^{-6}\times \left(2\cdot 4978^{-3}+20792^{-3}\right)\right)\right)}^{0.33333333}=630.02$ (error of *θ_D_* is 0.02 K or 0.00%);

$E\left(TiN_{0.67}O_{0.23}\right)$ $=5210\cdot 5747^{2}\left(3\cdot 9051^{2}-4\cdot 5747^{2}\right)$ $/\left(9051^{2}-5747^{2}\right)=399.99$ (error of *E* is 0.01 GPa or 0.00%),

$\theta_{D}\left(TiN_{0.67}O_{0.23}\right)$ $=479.92375477\cdot 10^{-13}$ $\times {\left(54.19927791\cdot 10^{23}/\left(146.96467178\cdot 10^{-6}\times \left(2\cdot 5747^{-3}+9051^{-3}\right)\right)\right)}^{0.33333333}=699.99$ (error of *θ_D_* is 0.01 K or 0.00%);

$E\left(TiN_{0.45}O_{0.28}\right)$ $=5080\cdot 4947^{2}\left(3\cdot 24321^{2}-4\cdot 4947^{2}\right)$ $/\left(24321^{2}-4947^{2}\right)=367.60$ (error of *E* is 2.40 GPa or −0.65%),

$\theta_{D}\left(TiN_{0.45}O_{0.28}\right)$ $=479.92375477\cdot 10^{-13}$ $\times {\left(54.19927791\cdot 10^{23}/\left(145.08183904\cdot 10^{-6}\times \left(2\cdot 4947^{-3}+24321^{-3}\right)\right)\right)}^{0.33333333}=629.06$ (error of *θ_D_* is 4.06 K or 0.65%);

$E\left(TiN_{0.22}O_{0.46}\right)$ $=5100\cdot 4751^{2}\left(3\cdot 23479^{2}-4\cdot 4751^{2}\right)$ $/\left(23479^{2}-4751^{2}\right)=340.44$ (error of *E* is 9.56 GPa or −2.73%),

$\theta_{D}\left(TiN_{0.22}O_{0.46}\right)$ $=479.92375477\cdot 10^{-13}$ $\times {\left(54.19927791\cdot 10^{23}/\left(143.67106374\cdot 10^{-6}\times \left(2\cdot 4751^{-3}+23479^{-3}\right)\right)\right)}^{0.33333333}=606.12$ (error of *θ_D_* is 16.12 K or 2.73%);

$E\left(TiN_{0.32}O_{0.40}\right)$ $=5080\cdot 4855^{2}\left(3\cdot 12960^{2}-4\cdot 4855^{2}\right)$ $/\left(12960^{2}-4855^{2}\right)=339.68$ (error of *E* is 10.32 GPa or −2.95%),

$\theta_{D}\left(TiN_{0.32}O_{0.40}\right)$ $=479.92375477\cdot 10^{-13}$ $\times {\left(54.19927791\cdot 10^{23}/\left(145.32685900\cdot 10^{-6}\times \left(2\cdot 4855^{-3}+12960^{-3}\right)\right)\right)}^{0.33333333}=612.55$ (error of *θ_D_* is 17.55 K or 2.95%);

$E\left(TiN_{0.36}O_{0.31}\right)$ $=5000\cdot 4790^{2}\left(3\cdot 12189^{2}-4\cdot 4790^{2}\right)$ $/\left(12189^{2}-4790^{2}\right)=323.21$ (error of *E* is 16.79 GPa or −4.94%),

$\theta_{D}\left(TiN_{0.36}O_{0.31}\right)$ $=479.92375477\cdot 10^{-13}$ $\times {\left(54.19927791\cdot 10^{23}/\left(145.44123810\cdot 10^{-6}\times \left(2\cdot 4790^{-3}+12189^{-3}\right)\right)\right)}^{0.33333333}=603.40$ (error of *θ_D_* is 28.40 K or 4.94%).

## Appendix B

The results of the verification and applicability of the Second algorithm for the TiN_*x*_O_*y*_ alloys

(on the example of the values for which the lowest errors were observed).

**Table:**

sample	*v*_s_	*v*_s_	Δ	*v*_m_	*v*_m_	Δ	λ_Cahill‒Pohl_	λ_Cahill‒Pohl_	Δ	*C*_p_	*C*_p_	Δ	*C*_V_	*C*_V_	Δ
	from table 5	by table 14	%	from table 5	by table 14	%	from table 9	by table 14	%	from table 9	by table 14	%	from Table 9	by table 14	%
TiN_0.24_O_0.88_	5736	5542	−3.38	6289	6128	−2.56	1.620	1.607	−0.80	41.022	41.013	−0.02	40.628	40.667	0.10
TiN_0.21_O_0.75_	5380	5559	3.33	5940	6143	3.42	1.587	1.611	1.51	38.750	38.622	−0.33	38.283	38.161	−0.32
TiN_0.20_O_0.65_	4799	4895	2.00	5474	5566	1.68	1.351	1.351	0.00	37.140	36.958	−0.49	35.845	36.167	0.90
TiN_0.21_O_0.62_	4781	4860	1.65	5457	5536	1.45	1.328	1.332	0.30	36.813	36.633	−0.49	35.495	35.746	0.71
TiN_0.30_O_0.54_	4975	5070	1.91	5609	5718	1.94	1.508	1.525	1.13	36.703	36.615	−0.24	35.894	35.722	−0.48
TiN_0.41_O_0.70_	5868	5699	−2.88	6405	6264	−2.20	1.653	1.628	−1.51	40.386	40.536	0.37	40.057	40.194	0.34
TiN_0.37_O_0.51_	5090	5175	1.67	5726	5809	1.45	1.529	1.539	0.65	37.097	37.093	−0.01	36.332	36.339	0.02
TiN_0.34_O_0.45_	4752	4878	2.65	5436	5552	2.13	1.156	1.183	2.34	35.843	35.774	0.19	34.384	34.566	0.53
TiN_0.57_O_0.56_	6012	5717	−4.91	6544	6280	−4.03	1.688	1.631	−3.38	40.577	40.537	−0.10	40.290	40.195	−0.24
TiN_0.56_O_0.55_	6012	5717	−4.91	6538	6280	−3.95	1.685	1.631	−3.20	40.257	40.250	−0.02	39.975	39.904	−0.18
TiN_0.44_O_0.36_	5030	4930	−1.99	5730	5597	−2.32	1.404	1.425	1.50	35.704	35.736	0.09	34.663	34.511	−0.44
TiN_0.52_O_0.34_	5351	5280	−1.33	5966	5901	−1.09	1.569	1.552	−1.08	36.368	36.502	0.37	35.835	35.572	0.73
TiN_0.51_O_0.27_	4978	4825	−3.07	5685	5506	−3.15	1.307	1.269	−2.91	35.204	35.297	0.26	34.034	33.863	−0.50
TiN_0.67_O_0.23_	5747	5612	−2.35	6320	6189	−2.07	1.627	1.618	−0.55	36.532	36.828	0.81	36.194	36.000	−0.54
